# A reinforcement learning-driven adaptive hybrid PLC-RF communication architecture for IoT-based smart metering systems

**DOI:** 10.1038/s41598-026-65952-0

**Published:** 2026-08-22

**Authors:** Noor El-Deen M. Mohamed, Mahmoud A. Shafea, Alaa M. Yousry, Mohamed M. El-Dakroury

**Affiliations:** 1 Computer and Systems Engineering Department, Faculty of Engineering, Capital University (formerly Helwan University), Cairo, Egypt; 2 Electronics and Communications Engineering Department, Faculty of Engineering, Capital University (formerly Helwan University), Cairo, Egypt

**Keywords:** Energy science and technology, Engineering, Mathematics and computing

## Abstract

With the rapid expansion of smart grid infrastructure, robust and scalable communication is essential to support Advanced Metering Infrastructure (AMI). This paper presents a hybrid communication architecture that combines Power Line Communication (PLC) and Radio Frequency (RF) technologies to enable reliable, adaptive data transmission in smart metering networks. The proposed system employs a PLC-based mesh topology that utilizes existing electrical wiring to relay meter data to a Data Concentrator Unit (DCU), alongside an RF-based star topology that provides direct wireless links between smart meters and the DCU. A cloud-based web application is integrated for real-time visualization of power consumption, network health, and active communication paths. To dynamically select the optimal channel based on real-time latency, packet loss, and Signal-to-Noise Ratio (SNR), a Proximal Policy Optimization (PPO) reinforcement learning agent is implemented and benchmarked against tabular Q-Learning, Double Deep Q-Network (DDQN), and Deep Deterministic Policy Gradient (DDPG). The system is validated through hardware prototyping and subjected to multi-seed training, reward function sensitivity analysis, edge microcontroller profiling, and network-scale simulation to assess deployment viability. Evaluated over 5, 000 deterministic test cases against a reward-derived oracle, the PPO agent achieved an accuracy of *93.78%* and an F1 score of *87.74%*, attaining the highest single-run performance among all evaluated agents, with statistically significant advantages over Q-Learning and, for F1 score, over DDPG. Multi-seed training across ten initializations confirmed superior convergence stability with an accuracy standard deviation of only *8.74%*, mitigating policy collapse. Edge AI profiling on an Arm Cortex-M4 platform demonstrated that INT8 quantization compresses the model by *2.36×* to 64.7 KB while preserving *>99.9%* of baseline accuracy at a 1.93 ms on-device latency. A reward sensitivity sweep across 16 coefficient perturbations verified policy robustness under varying weight configurations. Finally, a network-scale simulation across 500 to 5, 000 nodes confirmed scale-invariant performance with a Packet Delivery Ratio above *92%* and consistent resilience under node outages, traffic overload, and channel degradation, providing simulation-based evidence of the scalability and resilience of AI-driven adaptive communication for large-scale smart grid deployments.

## Introduction

The rapid deployment of high-end power systems has driven demand for intelligent and efficient energy management technologies, with Advanced Metering Infrastructure (AMI) emerging as a cornerstone of smart grid development. AMI enables utilities to perform real-time monitoring, control, and analysis of electricity usage. In this context, “real-time” refers to data collection at much higher frequencies (e.g., minutely or hourly intervals) than traditional monthly manual readings, resulting in enhanced operational efficiency, reduced energy losses, and improved demand response programs^1^. At the core of AMI lie smart meters, which serve as intelligent endpoints capable of capturing fine-grained consumption data and enabling two-way communication between consumers and utility providers. Recent reviews on smart meter data analytics, as reviewed in^2^, emphasize the critical role of leveraging massive volumes of meter data through descriptive, predictive, and prescriptive analytics to enhance grid efficiency, sustainability, and decision-making. From an Internet of Things (IoT) perspective, AMI can be viewed as a large-scale vertical IoT deployment, where smart meters act as resource-constrained edge devices connected through heterogeneous communication technologies to utility back-end and cloud services. A fundamental requirement for AMI’s success is a scalable and dependable communication network capable of interconnecting large numbers of geographically dispersed smart meters. Traditional solutions, Power-Line Communication (PLC) and Radio-Frequency (RF), each exhibit critical limitations: PLC suffers from noise and attenuation^3^, while RF is prone to interference and coverage gaps^4^. PLC leverages existing electrical wiring for data communication, offering cost-effective deployment, but endures significant signal attenuation, noise, impedance mismatches, and electromagnetic interference especially in legacy grids^3^. Galli et al.^3^ outlined the role of PLC in both in-home automation and utility-side grid monitoring, showing how it can enable widespread coverage without the need for additional cabling. However, PLC faces challenges including impulse noise, frequency-selective fading, and high channel attenuation due to varying electrical loads on the power lines. Challenges like impedance mismatches in PLC are addressed in recent tutorials on modem coupling circuits^5^, which highlight design trade-offs and safety considerations while providing insights into enhancing signal quality over low-voltage lines. Conversely, RF communication provides significant flexibility and ease of deployment. RF networks often utilize star topologies and are better suited for long-distance communication compared to PLC^6^. However, its performance can be adversely affected by external factors such as electromagnetic interference, physical obstructions, and limited coverage in densely populated or complex environments, as highlighted in^4^. To address these individual shortcomings and ensure robust coverage, hybrid approaches combining PLC and RF have gained significant attention. Avdaković et al.^7^ reviewed smart metering communication technologies across HAN, NAN, FAN, and WAN, highlighting the need for scalable, secure, and interoperable systems in low-voltage networks, which motivates hybrid PLC–RF solutions. Pittolo et al.^8^ showed that dynamic switching in hybrid PLC/Wi-Fi systems enhances robustness and throughput, supporting real-time adaptation in smart grids. Similarly, multi-interface nodes that switch between PLC and RF based on link quality provide resilient communication, while approaches like AAV-5G^9^ demonstrate that integrated wired–wireless architectures improve coverage and self-healing. These findings inform the proposed design of a PLC mesh combined with an RF star topology for reliable AMI data transmission.

### Related work

Two notable hybrid communication approaches in smart grids are G3-Hybrid^10^ and PRIME Hybrid^11^, both enhancing reliability and coverage by enabling seamless switching between PLC and RF. G3-Hybrid extends G3-PLC by unifying PLC and RF under a common MAC layer, selecting the channel per frame based on metrics like SNR, packet error rate, and routing cost, with retries over the alternate medium and deduplication to prevent redundancy. PRIME Hybrid integrates RF and PLC at the PHY layer with a shared MAC, dynamically selecting the medium using link quality indicators and thresholds, often leveraging IEEE 802.15.4 and CSMA/CA, while maintaining backward compatibility with PLC-only nodes^12^. Recent developments, such as those reviewed by Avdaković et al.^7^, highlight hybrid PLC/RF systems for smart grid monitoring, achieving up to 99% coverage in urban settings through interdisciplinary power and information engineering approaches. Similarly, Huc et al.^13^ provide a technical overview of G3-PLC Hybrid, demonstrating improved data rates and interference mitigation in field trials.

Early channel selection in hybrid systems often relied on threshold-based heuristics, which provide low-complexity switching without requiring data-driven training. For example, Bithas et al.^14^ propose an improved threshold scheme for wireless systems, applicable to PLC/RF hybrids, where channels are selected based on SNR or error rates falling below optimized thresholds. Using Markov chain analysis, it reduces complexity and outage probability by 20-30% compared to continuous monitoring, though it lacks adaptability to non-identical channel distributions common in dynamic grids. Critically, even adaptive threshold methods share a fundamental structural limitation: they optimize a single scalar trigger condition (e.g., SNR < $\gamma _{\text {th}}$) for each metric independently, and cannot simultaneously arbitrate among multiple correlated objectives such as packet loss, latency, and SNR under time-varying channel conditions. Furthermore, threshold schemes must be statically configured at deployment time and require expert re-calibration whenever traffic patterns, topology, or channel characteristics change, making them poorly suited to the heterogeneous and non-stationary PLC and RF environments observed in operational AMI networks.

Supervised learning (e.g., SVM) and semi-supervised approaches have been used to classify link quality, operating effectively when partial labels are available. However, training robust supervised models typically requires substantial labeled datasets, limiting their immediate plug-and-play use in hybrid PLC–RF systems due to the scarcity of real-time metric data like latency, packet loss, and SNR across diverse grid topologies. Moussa et al.^15^ compare ML models, including SVM with linear and non-linear kernels, for predicting PLC link availability in smart grids, achieving 85-86% accuracy on a 1,000-reading dataset of SNR, RSSI, and CINR. While these methods outperform simpler heuristics in noisy environments, the study highlights data correlation issues and the need for RF fallback, underscoring the challenges of applying purely supervised methods in highly variable scenarios without continuous retraining.

Beyond heuristic and supervised-learning-based schemes, recent work has explored the use of advanced routing and intelligence in AMI communication protocols. For instance, fault-tolerant routing schemes have been proposed to ensure reliable data aggregation in smart grid meshes^16^. Furthermore, reinforcement learning has been integrated directly into AMI routing, with a notable example being Q-RPL^17^, which integrates a Q-learning mechanism into the RPL routing protocol to optimize parent selection in wireless AMI mesh networks. By learning the next-hop choice based on link quality and routing performance, Q-RPL improves packet delivery ratio (PDR) by approximately 5–16% compared to standard RPL, RPL+, and ML-RPL variants, while achieving median end-to-end delays in the range of 177–286 ms and compliant factors of 94–100% in realistic smart meter deployments. This demonstrates that reinforcement learning can effectively enhance reliability and latency at the network (routing) layer of smart grid communication infrastructures. Unlike Q-RPL, which applies Q-learning at the routing layer to optimize parent selection in wireless AMI meshes, the proposed approach targets a complementary problem: node-level hybrid media selection between PLC and RF interfaces based on real-time link metrics. Rather than modifying the routing protocol, the proposed PPO agent operates as an interface-selection layer that learns when to use each medium while treating the underlying PLC and RF stacks as black boxes.

Reinforcement Learning (RL) offers a model-free alternative, learning policies through environmental interaction. As Gómez-Rodríguez et al.^18^ indicate, RL suits smart grids with unpredictable dynamics. A comprehensive review by Kim et al.^19^ on smart grid evolution and RL applications addresses challenges in power flow optimization and demand response, showing RL’s potential for scalable grid management. Similarly , a survey in^20^ regarding deep RL for smart grid operations highlights improvements in energy scheduling and fault resilience. For fault detection, Olojede et al.^21^ apply hybrid ML-RL models for predictive maintenance, achieving 88-93% accuracy in insulation fault detection. In real-time optimization for microgrids, Liu et al.^22^ use DDPG, reducing operating costs by up to 30% under uncertainties from renewables and loads. While Q-Learning suits discrete problems, extensions like DQN, DDQN, and DDPG enhance scalability, though PPO^23^ balances exploration and convergence, making it ideal for the dynamic hybrid networks.

More recently, the convergence of edge AI and IoT communication has produced several relevant research directions. Federated learning has been explored for privacy-preserving smart meter analytics, enabling distributed model training across AMI nodes without centralizing sensitive consumption data^24^. In the domain of lightweight embedded AI, TinyML frameworks have demonstrated the feasibility of deploying reinforcement learning agents on microcontrollers with as little as 32–512 KB of RAM through aggressive model compression techniques such as INT8 quantization, pruning, and knowledge distillation^25^. For hybrid communication reliability, recent analyses of smart grid communication infrastructure emphasize the importance of selecting optimal technologies across HAN, NAN, FAN, and WAN layers to ensure reliable, interoperable, and scalable data exchange^7^. These developments collectively inform the design choices in this work and highlight the growing maturity of AI-driven communication management in smart grid infrastructure.

The literature shows a clear trade-off: non-AI heuristic methods^14^ are simple but static, lacking the adaptability for dynamic grid conditions. Conversely, supervised ML models^15^ are data-driven but require large, pre-labeled datasets that are impractical for this application. This creates a research gap for a solution that is both adaptive and model-free. To summarize the trade-offs discussed, Table 1 provides a qualitative comparison of the different channel selection methodologies. This comparison highlights the gap addressed by this work, justifying the use of an RL-based approach over simpler, non-AI baselines.

**Table 1:** Qualitative comparison of channel selection and communication approaches.

Approach	Application	Adaptability	Data requirement	Complexity	Key limitation
Heuristic^14^	Wireless channel selection	Static (Fixed Rules)	Low (Manual Calibration)	Very Low	Fails in changing network conditions
Supervised & Semi-Supervised^15^	PLC node availability	Static (Post-Training)	High (Pre-Labeled)	Medium	Challenges obtaining diverse labeled datasets
Q-RPL^17^	AMI routing (network layer)	Dynamic (Learns)	Low (no pre-labeled)	Medium-High	Operates at routing layer, not physical
RL (This Work)	PLC/RF interface selection	Dynamic (Learns)	Very low	High	Advantage: Addresses data limitations

### Contribution

This paper proposes a hybrid PLC–RF communication system for smart metering networks, which dynamically selects the optimal communication channel based on real-time network conditions. The system integrates a PLC mesh topology, where smart meters relay data through neighboring nodes to a Data Concentrator Unit (DCU), providing enhanced coverage and fault tolerance. Simultaneously, an RF star topology implemented via the TI 802.15.4 stack^26^ that enables meters to transmit directly to an RF-enabled DCU, which subsequently forwards data to a cloud server for advanced processing and visualization.

While existing standards like G3-Hybrid and PRIME Hybrid provide mature dynamic switching capabilities at the MAC/PHY layers, the primary novelty and contribution of this work lies in the application and comparative analysis of four RL models (PPO, DDQN, DDPG, and Q-Learning) for intelligent interface selection. This work is distinct from existing approaches in the following respects. First, unlike G3-Hybrid and PRIME Hybrid, which perform threshold-based switching at the MAC/PHY layer using fixed link quality indicators, the proposed approach employs a learned policy that adaptively weights multiple channel metrics simultaneously without requiring manual threshold calibration. Second, unlike Q-RPL^17^, which applies Q-learning at the routing (network) layer to optimize parent selection in wireless meshes, this work targets a complementary and orthogonal problem: physical-layer interface selection between heterogeneous PLC and RF media. Third, unlike supervised learning approaches such as^15^, which require pre-labeled datasets and periodic retraining, the RL-based approach is model-free and learns directly from environmental interaction, making it suitable for deployments where labeled communication data is scarce. Instead of relying on static thresholds, the proposed PPO agent acts as an adaptive interface-selection layer that continuously evaluates real-time network performance metrics such as packet loss, latency, and signal-to-noise ratio (SNR) to dynamically decide the optimal medium. The models are trained in a novel simulation environment that is calibrated against empirical data from a physical hardware testbed. To validate real-world deployment feasibility, policy stability, and scalability, this work also introduces: (1) a multi-seed validation across ten distinct training seeds, verifying PPO’s superior convergence consistency and low variance (*8.74%* standard deviation in testing accuracy) against alternative off-policy algorithms; (2) edge hardware deployment profiling on an Arm Cortex-M4 microcontroller platform, where full INT8 quantization achieves a *2.36×* footprint compression (64.7 KB Flash occupancy) and an on-device latency of 1.93 ms while retaining *>99.9%* of the baseline accuracy; (3) a reward function sensitivity analysis under 16 parameter perturbations ($\pm 25\%$ and $\pm 50\%$ sweeps) showing PPO’s robustness to varying latency, loss, and switching cost weights; and (4) a simulation-based network-scale scalability and stress-testing evaluation across topologies of 500 to 5, 000 communication nodes, demonstrating scale-invariant Packet Delivery Ratio (PDR) above *92%*, low centralized batch latencies of 2.27 to 22.9 *μ*s, and strong resilience under node outages, traffic overload, channel degradation, and impulsive noise.

## System architecture

**Fig. 1:**
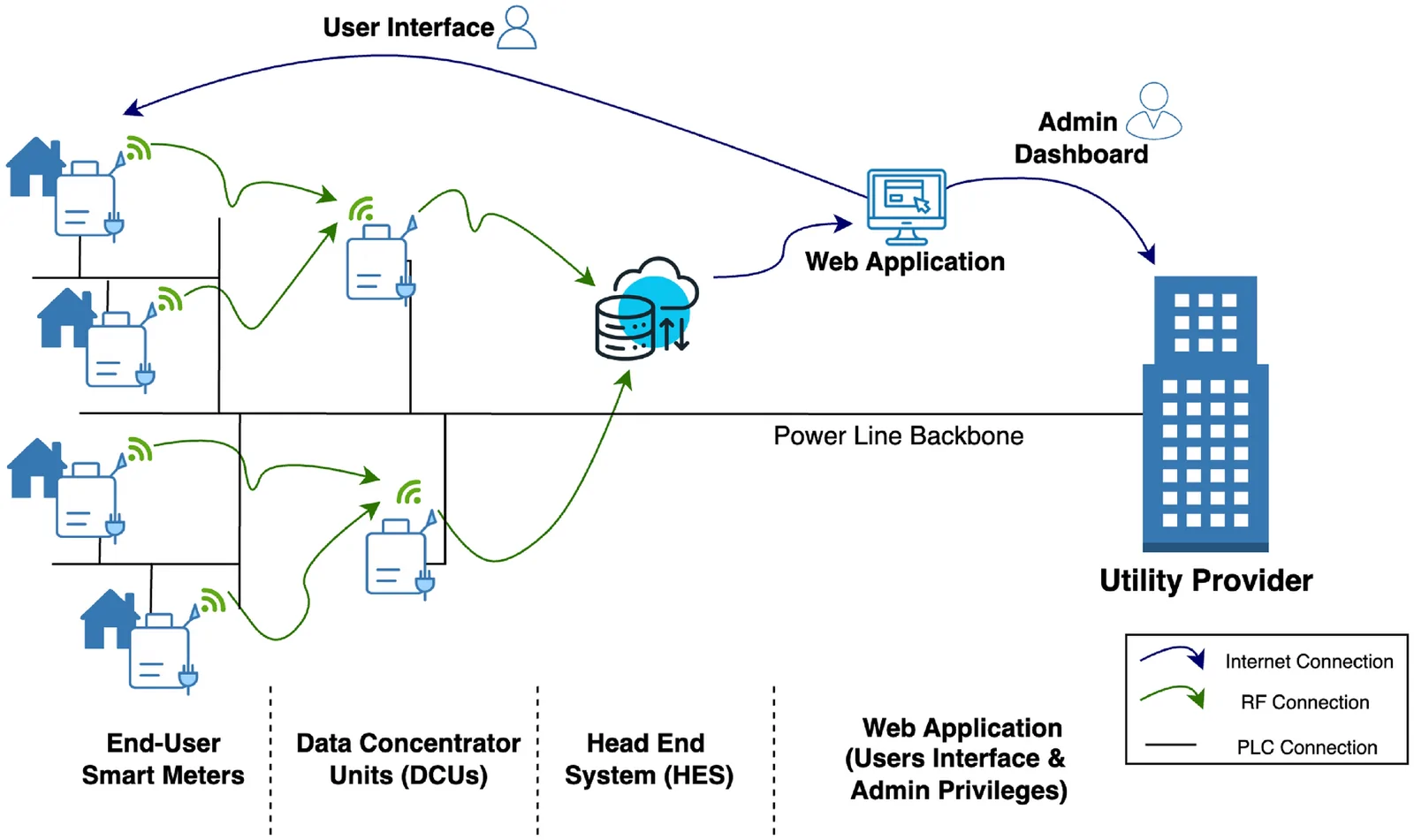
Hybrid communication system architecture.

The architecture in Fig. 1 enables reliable communication from residential smart meters to the utility provider under diverse channel conditions. This architecture reflects a realistic Advanced Metering Infrastructure (AMI) deployment, structured around four key components: end-user sensor nodes (smart meters), Data Concentrator Units (DCUs), the utility’s Head-End System (HES), and the cloud-based web application. Together, these components form a hybrid communication pipeline that supports real-time data collection, aggregation, management, and centralized analytics. Although quantitative scalability limits depend on the deployment environment, standard configurations support multiple PLC hops and extended RF ranges under typical loads, establishing a robust framework for large-scale AMI. Each end-user sensor node is installed at the consumer’s premises and serves as the first point of data generation. These nodes are equipped with a dual-mode communication system that supports both power line communication (PLC) and sub-GHz radio frequency (RF) transmission. The end-user nodes and DCU components in the proposed architecture leverage principles from model-based safe RL for distribution systems^27^, which demonstrate how reinforcement learning can be constrained to guarantee operational safety while optimizing system performance. By analogy, the proposed PPO-based framework dynamically switches between PLC and RF communication paths, ensuring consistent data transmission under varying interference and signal conditions while avoiding unsafe or unstable routing decisions. The hardware stack comprises an STM32F401RCT6 microcontroller for onboard data processing and control operations^28^, a TI1352B1 RF module operating on the TI 15.4 software stack for IEEE 802.15.4g-compliant wireless communication^29^, and an LX200V50 PLC module for data transfer over the electrical grid infrastructure^30^. Additionally, each node is embedded with a lightweight AI agent based on the Proximal Policy Optimization (PPO) reinforcement learning model. This agent dynamically selects the most reliable communication path in real time by evaluating environmental metrics such as signal-to-noise ratio (SNR), packet loss, and latency. The hybrid design not only enhances communication reliability but also improves system continuity by allowing intelligent switching between RF and PLC depending on link quality.

Data generated by the sensor nodes after getting it from the smart meters at homes is transmitted through two primary communication pathways: PLC and RF. In the PLC mode, data is sent over the existing electrical wiring to a nearby Data Concentrator Unit (DCU). While PLC is cost-effective and utilizes pre-existing infrastructure, it is susceptible to noise, impedance mismatches, and signal attenuation especially in legacy electrical environments. These challenges necessitate the use of effective routing protocols and error correction techniques. Conversely, the RF path relies on low-power wireless transmission using the 802.15.4g standard. In this mode, multi-hop communication is employed, where data is relayed through intermediate nodes until it reaches the Data Concentrator Unit (DCU). This wireless channel acts as a redundant path, ensuring communication continuity in scenarios where PLC links are degraded or interrupted. To optimize performance under changing network conditions, an adaptive channel selection strategy is implemented using the PPO model embedded in each sensor node. This model continuously monitors real-time metrics and autonomously selects the most suitable channel for data transmission. Compared to traditional reinforcement learning approaches, including Q-Learning or DDPG, PPO demonstrates more stable convergence, improved exploration–exploitation balance, and greater robustness in highly variable environments . The Data Concentrator Units (DCUs) aggregate data from end-user sensor nodes within their area. These DCUs receive data via both RF and PLC links, buffer the information, and then utilize a high-bandwidth backhaul (such as cellular or Ethernet) to transmit the aggregated data to the utility’s central Head-End System (HES). Strategic placement of these DCUs, often downstream from a distribution transformer, ensures full coverage for a specific neighborhood or low-voltage segment, guaranteeing consistent data flow. All collected data from across the AMI network converges at the HES, which handles data validation, storage, and management. The proposed cloud-based web application then acts as the central intelligence and visualization layer by interfacing with the HES. This platform pulls the managed data to provide dashboards for both end users and utility administrators. Users can view real-time energy consumption and historical trends, while administrators are given access to advanced system controls, anomaly detection tools, and maintenance alerts. The web application backend is designed for deployment on a secure cloud infrastructure and supports explicit role-based access control (RBAC), billing analytics, and fault management features. This RBAC implementation ensures end-users see only their own data, while utility administrators access system-wide diagnostics and controls. A comprehensive security analysis is essential in AMI deployments to mitigate risks such as PLC eavesdropping, potential RF jamming attacks, and adversarial perturbations targeting the reinforcement learning agent’s observation space. While a full exploration of these cryptographic and adversarial robustness requirements is beyond this paper’s immediate scope, the proposed architecture assumes standard security protocols (e.g., TLS/SSL for backhaul, AES encryption at the physical/MAC layers) are enforced, and future extensions will investigate adversarial training to fortify the PPO agent in real-world smart grid contexts.

## Hybrid IoT communication approach

**Fig. 2:**
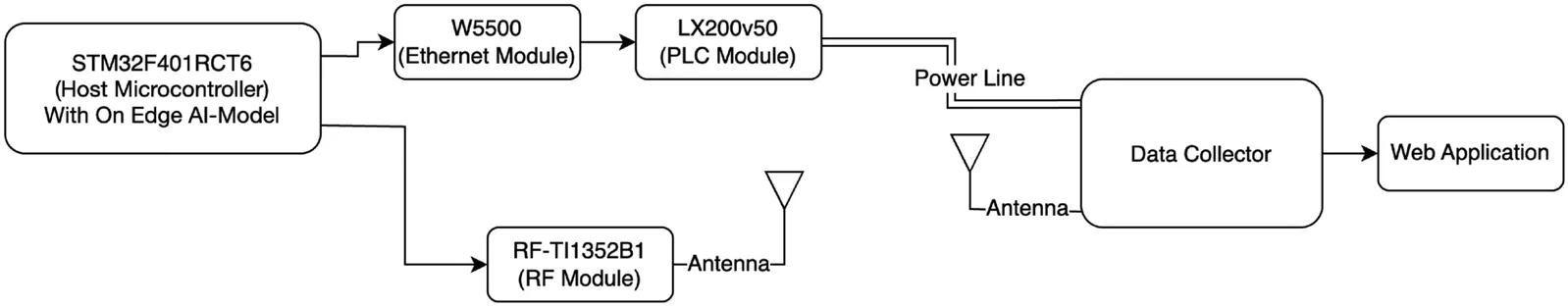
Hardware architecture.

As depicted in Fig. 2, the system integrates embedded modules for sensing, processing, and communication, while also deploying logical networking overlays and protocol stacks to support stable operation across wired and wireless channels. Key software integrations include the TI 15.4 stack for RF communication and a VPN-based solution for creating a logical mesh network over the PLC infrastructure. Additionally, this section outlines the technical decisions behind hardware–software compatibility, the configuration of network roles, and the performance validation setup. The overall system is designed to maintain high data availability and recover from link failures by intelligently switching between communication mediums based on real-time network conditions.

### RF network

**Fig. 3:**
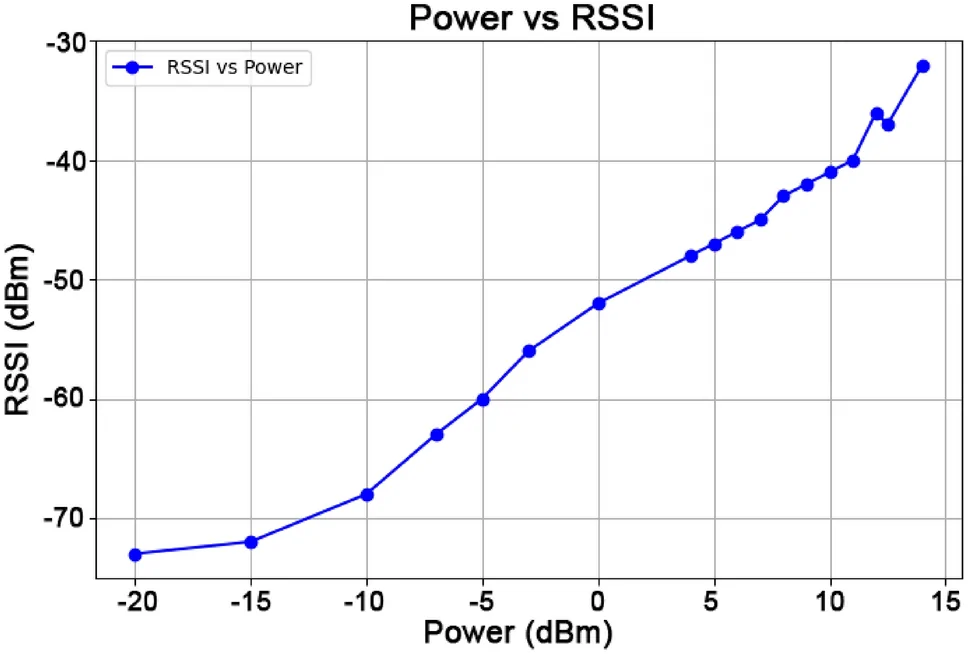
RSSI level corresponding to tested power.

The RF communication channel was thoroughly tested to validate its performance, connectivity, and seamless integration with the central DCU and backend infrastructure. The setup employed TI CC1352R modules operating under the TI 15.4 stack, configured in a star topology, as previously described. To assess the signal quality and transmission range, a controlled point-to-point test was conducted between two RF nodes in a typical indoor lab environment at a varying distance of 0.5 meters to 1.0 meters. In this experiment, one node continuously transmitted data packets while the receiving node measured the Received Signal Strength Indicator (RSSI) at varying power levels. The evaluation, illustrated in Fig. 3, shows the relationship between transmission power and the resulting RSSI. While higher power levels did yield marginal RSSI increases, the performance gains plateaued significantly after 10 dBm. Therefore, the 10 dBm level was selected as the optimal operating point. This power level provides a strong RSSI (approx. -40 dBm), ensuring a reliable link margin, while avoiding the diminishing returns and unnecessary energy consumption of operating at maximum power.

Following the initial range validation, three sensor nodes were deployed, each built around an STM32F401RCT6 microcontroller connected to a CC1352R RF module. These nodes were configured as child devices under the TI 15.4 protocol stack and successfully established connections with a single RF DCU node. The sensor nodes were programmed to periodically transmit structured packets containing unique sensor identifiers and simulated (dummy) data values. The DCU’s monitor showed the correct number of joined devices (3), which confirmed successful reception of the transmitted packets, and then it forwarded the aggregated data to the cloud-based web server for further processing and visualization. This test validated end-to-end communication, demonstrating the correct functioning of both RF data acquisition and backend data integration.

### PLC network

**Fig. 4:**
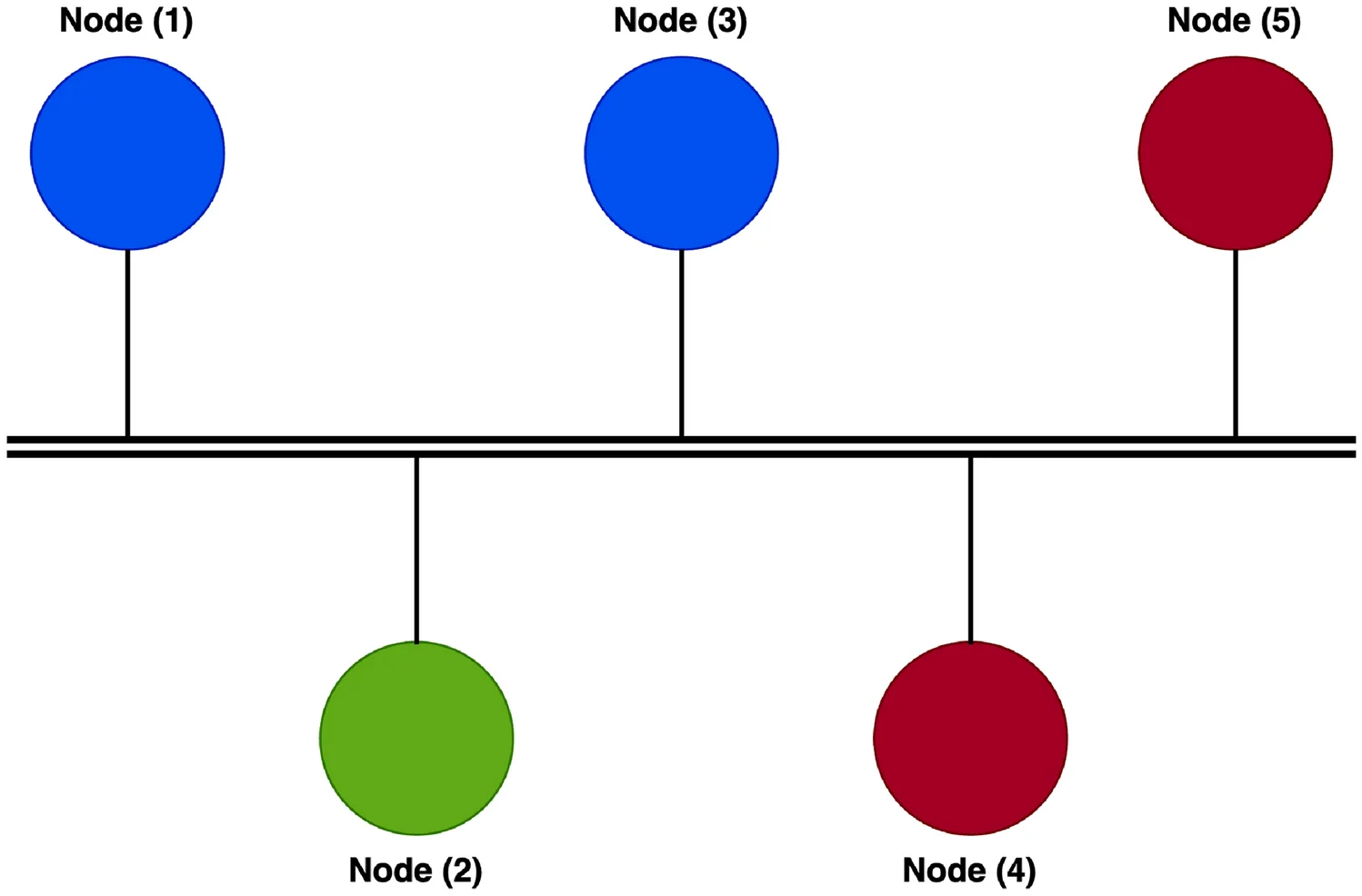
PLC physical topology.

For the PLC network, a mesh topology was logically implemented to allow flexible multi-hop data forwarding among smart meters. However, it is important to note that the physical topology as illustrated in Fig. 4 remains a bus configuration, as all nodes share a single electrical backbone originating from the utility provider’s power line, and then different nodes connect to it. This structure enables cost-effective deployment by leveraging existing infrastructure, while the mesh logic ensures improved connectivity and adaptability in data routing, particularly in scenarios with disrupted direct communication paths. Although the physical topology of the PLC network follows a bus configuration, with all nodes connected to a single power line backbone from the utility provider, it operates as a logical mesh. In this design, each node is aware of its peers and can establish direct or indirect communication paths as needed. This is especially beneficial for long-range deployments spanning several kilometers, where some nodes may face signal degradation or be unable to reach the destination directly. Multi-hop forwarding addresses this by allowing packets to be relayed through intermediate nodes until they reach the DCU. As a result, the logical mesh significantly improves communication success rates and link redundancy in practical PLC environments.

The PLC network was validated in two phases using commercially available LX200V50 modules as a proof of concept to test its logical capabilities. First, a point-to-point (P2P) test was conducted to establish a stable communication range, which was determined to be approximately 10 meters within the laboratory environment. Second, a 5-node logical mesh test was performed to validate the system’s functional operations, such as the VPN overlay and fault-tolerant rerouting. In this test, five PCs simulated smart meter nodes on the same electrical phase, with nodes spaced approximately 1.2 meters apart and a 2-meter separation between Node3 and Node4. This configuration was designed to verify the mesh’s logical self-healing capabilities, which are distinct from the physical signal performance over a large-scale distribution network. A VPN overlay created a logical full mesh, allowing secure, direct communication between all nodes. To test fault tolerance, the direct logical link between Node1 and Node3 was administratively disabled, demonstrating the system’s ability to autonomously reroute traffic through alternative paths (via Node2, for example).

**Fig. 5:**
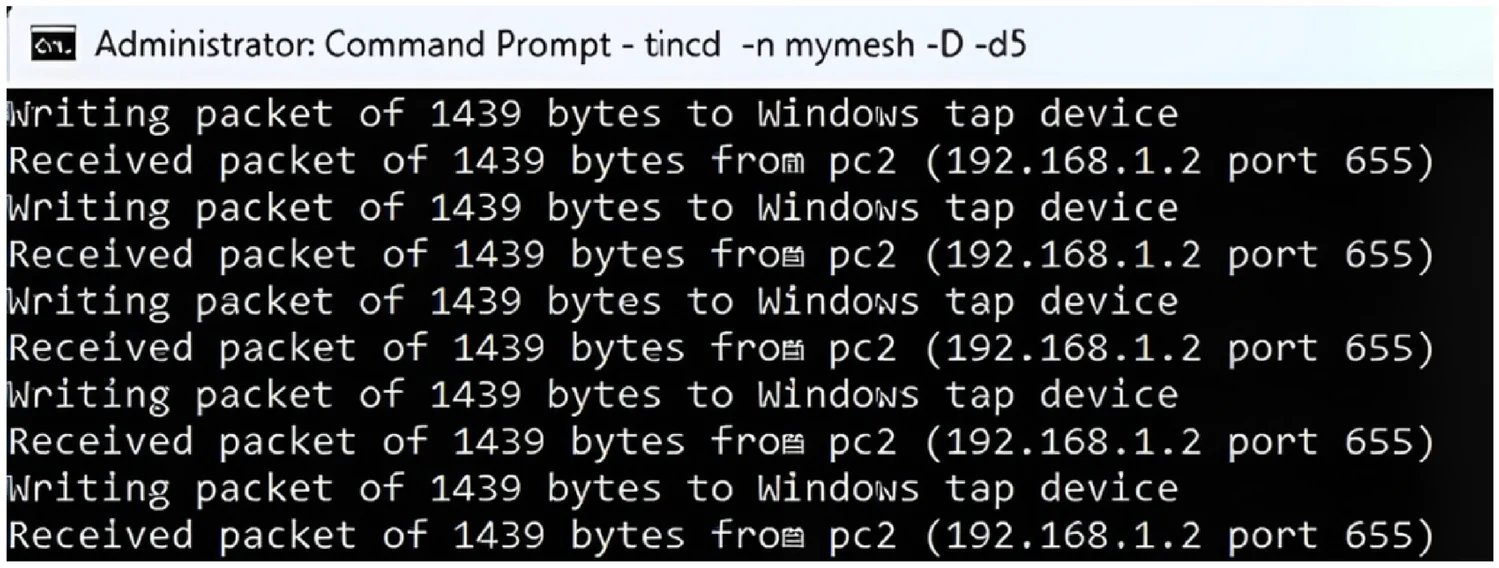
Validating data forwarding from PC2.

However, with the link disabled, the system relied on the VPN’s dynamic routing capabilities, which successfully rerouted traffic through PC2. This intermediate relay behavior was confirmed using network monitoring tools, as shown in Fig. 5, where relayed packets were observed on PC2. The successful forwarding of data in the absence of a direct path demonstrated the self-healing nature of the logical PLC mesh network, validating its suitability for smart metering applications in variable and failure-prone electrical environments.

Following the successful topological validation of the PLC mesh, the network’s link quality was assessed using key performance metrics such as latency, packet loss, and throughput. These measurements were collected across different PC pairs within the mesh under typical operating conditions. The evaluation aimed to quantify the communication reliability and efficiency of the LX200V50-based PLC links under mesh routing. The results, summarized in Table 2, provide insight into the variability of link performance and the effectiveness of the mesh topology in maintaining stable data transmission across diverse electrical paths.

**Table 2:** PLC mesh test results.

Test	Bitrate (Mbits/sec)	Jitter (ms)	Lost/Total datagrams	Loss (%)
Test 1	21.4	1.812	17,955 / 134,203	13
Test 2	22.3	0.445	14,362 / 134,195	11
Test 3	23.9	0.871	5,558 / 134,214	4.1

While this initial point-to-point and mesh evaluation recorded packet losses between 4% and 13%, it is important to note that these figures stem from prototype implementation overheads in early firmware rather than fundamental media limits, and would typically be mitigated in a production AMI setup (which requires >99% reliability) through ARQ mechanisms, robust error-correction coding, and optimized hardware drivers. Nonetheless, this test validates the logical feasibility and self-healing nature of deploying PLC as a communication backbone in a distributed mesh network.

## Web application

To facilitate centralized monitoring, diagnostics, and control of the hybrid communication system, a full-stack web application was developed using scalable and production-grade technologies. The backend layer was built using Node.js^31^, a non-blocking, event-driven runtime environment known for its efficiency in handling concurrent data streams with low latency overhead. The Express.js framework^32^ was employed to structure the backend into modular RESTful APIs, responsible for managing authentication, role-based authorization, and interfacing with the HES to manage data. The modular design enables the platform to efficiently process continuous smart meter data while remaining maintainable and extensible, decoupling ingestion, storage, analytics, and visualization to optimize performance. This architectural approach draws modular inspirations from recent real-time IoT data processing frameworks^33^, while avoiding the unnecessary communication overhead of a full distributed microservices model. This design supports real-time event-driven processing of PLC and RF data while ensuring interoperability and scalability for integration with future services such as predictive analytics and grid-wide monitoring. Data persistence and retrieval were handled using MongoDB Atlas^34^. On the client side, the interface was built using React.js^35^, a declarative JavaScript library for building interactive and component-based UIs. The frontend provides separate views for different user roles. For example, end-users are presented with energy consumption summaries and trend visualizations, while utility administrators and technicians have access to detailed network diagnostics and AI decision logs (which record the channel chosen dynamically by the on-edge AI agent, the network metrics of that time). Real-time visualizations are powered by integrated charting libraries that render consumption data, signal strength trends, and packet loss heatmaps. A notable feature of the application is the network map, which displays the active communication path (PLC or RF) used by each smart meter in real time. This visualization reflects the decisions made by the PPO-based AI model and helps operators understand system behavior and resilience. The web application’s design also includes a framework for notification systems to alert users to abnormal events such as persistent communication failures, unexpected usage spikes, or performance degradation allowing timely intervention. System validation included confirming that the data acquired by the RF DCU, as there are three instances of receiving reading requests, each one from a different sensor, was accurately transmitted to the backend server and correctly rendered on the web application’s dashboard for real-time visualization. This confirmed the end-to-end integrity of the RF communication pathway spanning node-level transmission, centralized data aggregation, backend processing, and real-time visualization through the web interface.

### User dashboard

The MeterFlow user dashboard provides a dynamic view of real-time energy usage. Data received via the PLC or RF channel is immediately processed by the backend server and reflected on the dashboard, enabling users to monitor consumption patterns and key metrics. The system also offers a Consumption page that details electricity usage and meter status, including Meter ID, location, and PLC metrics such as latency and packet loss. Configurable thresholds for warning and critical consumption levels allow users to proactively manage their energy usage.

### Admins dashboard

**Fig. 6:**
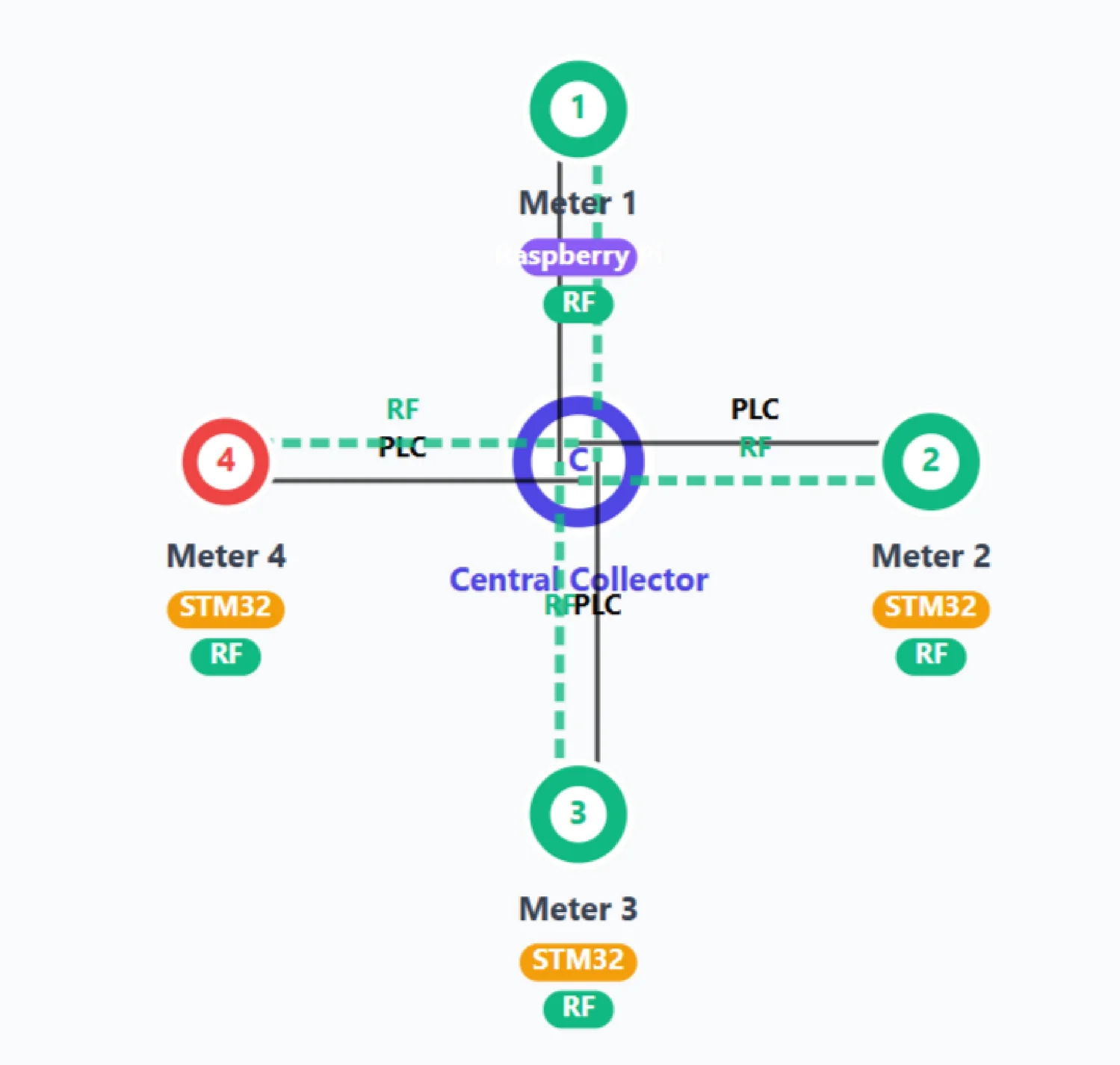
Network map showing active links.

The MeterFlow web application offers an administrator dashboard for system-level monitoring and control, presenting aggregated metrics such as total meters, overall energy consumption, and pending notifications. It includes panels for Meter Status (Online, Maintenance, Offline) and Communication Lines (PLC vs. RF), enabling utility providers to assess real-time system performance across the infrastructure. The network map, shown in Fig. 6, highlights the communication channel selected by the local PPO agent embedded in each meter, using distinct node colors (blue for the Central DCU, green for online meters, red for offline) and line styles (solid black for PLC, dashed green for RF). This visualization provides a real-time geographic map of the network, displaying the active communication channel (PLC or RF) and operational status (online, offline) for each meter. This allows operators to identify network performance patterns and correlate them with physical locations for maintenance and optimization.

## Reinforcement learning for channel selection

Integrating Artificial Intelligence (AI) into a hybrid PLC–RF communication enables adaptive channel selection based on real-time network conditions. Such intelligent switching is vital for improving data integrity (minimizing packet loss) and timeliness (reducing latency) in smart metering and industrial automation environments. This work focuses on developing a reinforcement learning-based channel selection mechanism for hybrid PLC–RF communication under dynamic conditions.

Threshold-based methods in G3-Hybrid face three limitations that threshold optimization alone cannot resolve: (i) they cannot jointly optimize over correlated metrics (e.g., high SNR but elevated packet loss simultaneously), (ii) they degrade under non-stationary channel statistics that shift outside the calibrated operating range, and (iii) they do not generalize across heterogeneous PLC and RF channel characteristics without per-deployment re-tuning. A PPO-based policy, by contrast, learns a non-linear decision boundary across the full joint metric space from experience and updates through gradient-based optimization, enabling adaptation to channel dynamics that invalidate fixed thresholds. Furthermore, the proposed interface-selection layer operates entirely above the MAC sublayer, acting on link-quality observations exposed by the existing G3-PLC Hybrid and TI 15.4 stacks without modifying their internal frame scheduling, retransmission, or deduplication mechanisms. This means the architecture is fully coexistent with standard-compliant G3-Hybrid or PRIME Hybrid MAC implementations and does not require a replacement protocol stack.

### Methodological framework

A key challenge in developing such a mechanism is the lack of publicly available, labeled datasets that capture comprehensive metrics (e.g., latency, packet loss, SNR) across diverse PLC and RF environments. This data scarcity makes traditional supervised learning approaches impractical. To overcome this, a Reinforcement Learning (RL) approach was adopted. RL provides a model-free, data-efficient alternative by letting an agent learn a policy through direct interaction with the environment. To identify the most effective strategy for this dynamic channel selection problem, four distinct RL algorithms were implemented and compared: Q-Learning, as a foundational, tabular-based baseline to assess performance in a discretized state space; Double Deep Q-Network (DDQN), chosen for its ability to handle continuous state spaces using function approximation and for its known stability improvements over standard DQN; Deep Deterministic Policy Gradient (DDPG), selected as a state-of-the-art actor-critic method for continuous action spaces, adapted here for a binary choice; and Proximal Policy Optimization (PPO), implemented due to its strong reputation as a policy-gradient method that balances exploration, convergence speed, and stable performance, making it a suitable candidate for dynamic network environments. This comparative approach enables a robust evaluation of trade-offs between model complexity and decision performance in hybrid communication.

The selection of Q-Learning, DDQN, DDPG, and PPO as the four evaluated baselines was guided by the specific structural properties of the channel selection problem rather than an attempt to survey the full space of modern reinforcement learning algorithms. The task addressed in this work is a single-agent, binary discrete-action decision made independently at each node from a low-dimensional, memoryless six-dimensional state, as established in “Simulated training environment” section. The four chosen algorithms span the principal methodological axes relevant to this setting: tabular value-based learning through Q-Learning, deep value-based learning with function approximation through DDQN, continuous-action actor-critic learning adapted to a binary decision through DDPG, and clipped-objective policy-gradient learning through PPO. This spread allows the comparison to isolate whether representation capacity, learning paradigm, and update stability mechanism drive performance differences on this task, which is the central methodological question addressed by the testing and multi-seed evaluations in “Testing and evaluation” and ’Statistical significance and multi-seed validation” sections.

More recent algorithms noted by the reviewer target problem characteristics that are not present in the current formulation. SAC and TD3 extend the continuous-control paradigm already represented here by DDPG through additional entropy regularization or twin critics, improvements that primarily address continuous action spaces rather than the binary PLC or RF decision studied here. DDPG was retained specifically to test whether a continuous-action actor-critic method transfers to this discrete setting, and the results in “Statistical significance and multi-seed validation” and “Reward function sensitivity and policy robustness analysis” sections show pronounced instability, with a mean F1 score as low as 13.03 percent in multi-seed evaluation and policy collapse under reward coefficient perturbations, providing empirical rather than assumed justification for this choice. Rainbow DQN combines several discrete value-based refinements, most of which target large discrete action spaces such as those in Atari-scale benchmarks; the two-action space evaluated here already isolates the double Q-value correction through DDQN, so the incremental contribution of the remaining Rainbow components is expected to be small. A3C and IMPALA are primarily distributed training architectures developed to accelerate learning through many parallel environment instances; the present environment converges within 200 to 300 episodes, as reported in “Training and hyperparameters” section , so distributed training infrastructure offers no meaningful benefit at this scale. Transformer-based reinforcement learning is designed to model long-range temporal dependencies in sequential or partially observable tasks, whereas the PLCRFNetworkEnv simulation environment is explicitly memoryless, with channel metrics re-sampled independently at every step as detailed in “Simulated training environment” section , removing the sequential structure that transformer-based architectures are built to exploit. Graph reinforcement learning and multi-agent reinforcement learning are suited to problems requiring coordination across an explicit network topology; the present architecture instead performs independent, per-node inference with no inter-node coordination, a deliberate design choice discussed in “Scalability and deployment considerations” section that keeps the per-node computational and communication overhead constant as the network scales. Extending the comparison to a coordinated multi-agent or graph-based formulation is acknowledged as a promising direction for future work. A concrete motivating scenario for such coordination is the DCU-level correlated impulsive noise event modeled in “Simulation-based network scalability and stress testing” section: when all nodes within an affected DCU cluster experience degraded PLC quality simultaneously, independent per-node policies may each individually switch to RF at the same time, concentrating traffic and increasing the risk of the RF congestion already observed in the high-traffic stress scenarios in “Simulation-based network scalability and stress testing” section. A multi-agent framework in which neighboring meters share local channel-quality information, or in which a DCU-level coordinator arbitrates channel assignment across its cluster, could in principle balance channel load during such correlated events rather than allowing every node to react identically and independently, and represents a promising extension of the per-node decision framework studied in this work.

### Simulated training environment

In RL, the environment defines the context for learning. A custom environment, ‘PLCRFNetworkEnv‘, was developed to simulate the two-channel (PLC and RF) communication system. The agent’s objective is to select the optimal channel on a per-packet basis. Each state is a 6-dimensional vector containing three features for each channel: latency, packet loss, and Signal-to-Noise Ratio (SNR). The agent has a discrete action space with two choices: select the PLC channel (Action 0) or the RF channel (Action 1).

The three-metric state representation was designed to capture the essential and orthogonal dimensions of link quality for the binary PLC/RF interface selection task. Latency characterizes access delay, packet loss characterizes link reliability, and SNR characterizes channel quality; together they provide a complete description of whether a communication path is suitable for AMI data delivery at the packet level. The strong classification performance achieved by PPO (93.78% accuracy, 87.74% F1-score) and the reward sensitivity analysis, which shows policy stability under $\pm 50\%$ perturbations of each individual metric weight, both confirm that this representation is sufficient to learn a robust and well-generalizing channel selection policy. Extending the state with additional indicators such as RSSI, LQI, BER, queue occupancy, or residual energy is a natural and promising direction for future work, as a richer state space could enable finer-grained decisions in more complex multi-hop or heterogeneous deployments. Directionally, indicators such as BER, RSSI, and LQI are expected to refine rather than qualitatively change the current decision boundary, since they largely provide lower-level confirmation of the same channel-quality dimension already captured jointly by SNR and packet loss, with the largest expected benefit concentrated in marginal-SNR conditions where the coarser three-metric state may be ambiguous. Queue occupancy, by contrast, would expose congestion state before it manifests as packet loss, and could be expected to reduce reactive switching frequency under the traffic-overload conditions evaluated in “Simulation-based network scalability and stress testing” section by allowing the agent to anticipate congestion rather than respond to it after the fact. Residual node energy would allow the policy to weigh the fixed RF-preference bonus in the reward function defined in “Reward function design” section against the actual battery state of each node, which would be expected to shift channel selection toward PLC for energy-depleted nodes even when RF offers a marginal link-quality advantage. For the present binary selection problem, however, the three chosen metrics provide a compact, hardware-native, and proven-sufficient basis that also keeps the policy network within the 64.7 KB Flash budget required for embedded inference on the Cortex-M4 microcontroller.

A primary challenge in simulated training is the gap between simulation and real data. To ground the simulation in a realistic environment and ensure its foundation is based on real-world data, the simulation parameters were calibrated using empirical data collected from the hardware testbed. While this logged data provided the statistical basis for the simulation, a static dataset is insufficient for training an RL agent, which must be able to actively explore the environment to learn an effective policy. The calibrated simulation provides a safe and scalable environment for this exploration, allowing the agent to learn from a wide variety of channel conditions, including rare events not captured in the limited hardware logs.

**Fig. 7:**
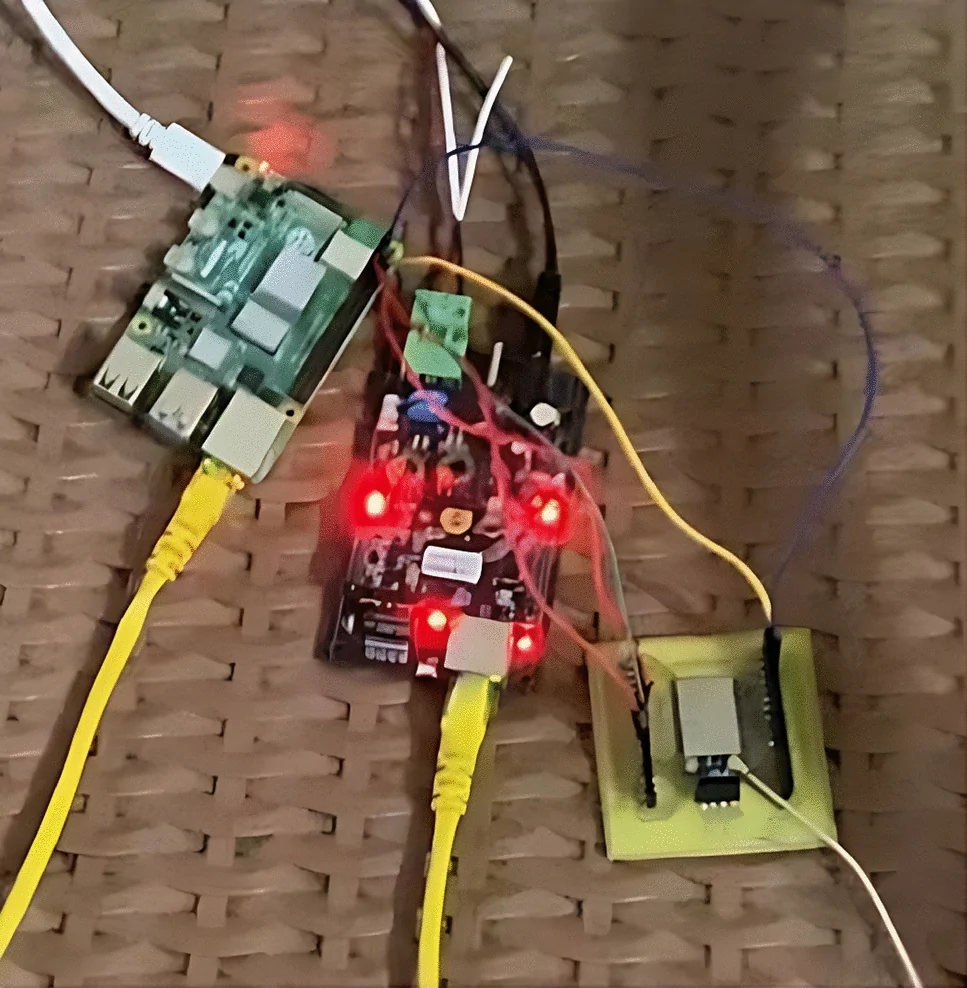
The hardware testbed used for validation, showing the system running on a Raspberry Pi 4.

Five distinct test scenarios were conducted across multiple environments, including a personal residence and a research lab, with varying electrical loads on the PLC network. During these tests, the performance of the LX200V50 PLC modules and TI CC1352R RF modules was logged. The hardware testbed used for validation is shown in Fig. 7, with the PPO agent running on a Raspberry Pi 4. The aggregated, observed ranges from these hardware logs are summarized in Table 3.

**Table 3:** Empirically observed data ranges from hardware tests.

Metric	PLC (LX200V50)	RF (TI CC1352R)
Latency (ms)	0.5–1.5	10.0–25.0
Packet Loss (%)	0.0–10.0	0.0–8.0
SNR (dB)	3.0–12.55	50.0–90.0

The simulation parameters in the ‘PLCRFNetworkEnv‘ were then set to match these observed characteristics. For instance, ‘self.packet_loss_plc_max‘ was set to 0.10 (10%) and ‘self.latency_rf_mean‘ was set to 16.0, directly reflecting the empirical data from Table 3. This calibration ensures the agent is trained in an environment statistically representative of the target deployment and grounded in real hardware data.

To support reproducibility, Table 4 summarises the per-metric sampling distributions used in PLCRFNetworkEnv, with all parameters calibrated directly from the hardware-observed ranges in Table 3. The following qualitative assumptions also apply. The environment is memoryless: channel metrics are re-sampled independently at every time step, so no temporal correlation or fading memory is modeled. No explicit multipath fading, cyclostationary PLC noise, or co-channel RF interference model is applied; the empirical bounds on packet loss and SNR implicitly capture the aggregate effect of these impairments as measured across five hardware test scenarios at varying electrical loads. All nodes are static, consistent with the fixed-infrastructure AMI deployment scenario. The environment does not model packet queuing or a stochastic arrival process; each discrete time step represents one channel-selection decision, abstracting the packet-level transport layer. The environment is fully synchronous; at each step the agent observes the current 6-dimensional state vector, selects an action, and receives an instantaneous scalar reward with no propagation delay modeled. The state vector is min-max normalized to [0, 1] before being passed to the agent. Each episode consists of 10 sequential time steps; new channel conditions are independently re-sampled at every step and at each episode reset, ensuring statistically independent episodes. The global random seed is fixed to 42 for single-run evaluations and varied across ten distinct seeds for the multi-seed analysis, enabling exact reproducibility of all reported results. The minimum and maximum bounds of each sampling distribution in Table 4 directly correspond to the best-case and worst-case values recorded across the five hardware test scenarios described above, so the range of channel severity observed in practice, including comparatively poor conditions encountered during testing, is represented within the calibrated bounds even though the base environment does not model the temporal clustering of such conditions. Burst-type degradation is evaluated separately at network scale through the correlated impulsive noise mechanism described in “Simulation-based network scalability and stress testing” section , where the PPO policy Packet Delivery Ratio drops by less than one percentage point under bursty PLC noise relative to clean conditions, as reported in the stress-test results. Correlated RF fading specifically, however, has not been modeled at either the base environment or network-scale level, since RF channel samples remain independently generated at every step in both cases.

**Table 4:** Simulation environment sampling distributions for PLCRFNetworkEnv.

Metric	PLC	RF
Latency (ms)	$\mathcal {N}(1.0,\,0.5^2)$, clipped *[0.5, 1.5]*	$\mathcal {N}(16.0,\,3.0^2)$, clipped *[10.0, 25.0]*
Packet Loss	$\mathcal {U}(0.0,\,0.10)$	$\mathcal {U}(0.0,\,0.08)$
SNR (dB)	$\mathcal {U}(3.0,\,12.55)$	$\mathcal {N}(70.0,\,10.0^2)$, clipped *[50.0, 90.0]*

### Algorithm implementation

To learn the optimal channel selection policy, four distinct reinforcement learning algorithms were implemented and compared. Each algorithm explores the simulated environment and attempts to learn a policy that maximizes a cumulative reward signal. The specific definition of this reward, which forms the core of the agent’s decision-making logic, is detailed in the following section. The implemented algorithms are as follows:

Q-Learning. The Q-Learning model follows a standard tabular approach, maintaining a Q-table to learn the action-value function Q(s,a). This function estimates the expected future reward for taking an action (e.g., choose PLC) from a given state (the current channel metrics). To use a tabular function with continuous inputs, the 6-dimensional state space is discretized into fixed bins, forming a unique, compact state representation. Action selection is governed by an *ε*-greedy strategy, a standard method to balance exploration (random actions) and exploitation (choosing the action with the highest Q-value)^36^. The learning rate *α* and discount factor *γ* control the influence of new information and the valuation of future rewards, respectively. This lightweight implementation serves as an interpretable baseline.

Double Deep Q-Network (DDQN). To mitigate the overestimation bias of standard Q-learning and handle the continuous state space directly, the Double Deep Q-Network (DDQN) algorithm was implemented. DDQN uses two neural networks: an online network for action selection and a target network for evaluating Q-values. The model takes the 6-dimensional state vector as input and outputs Q-values for each action. Its architecture consists of three fully connected hidden layers (256, 256, 128 neurons) with ReLU activation. Training utilizes a replay buffer and the Adam optimizer. The target Q-value is computed using the DDQN update rule:

1 $$\begin{aligned} Q_{\text {target}}(s_t, a_t) = r_t + \gamma \cdot Q_{\text {target}}\left( s_{t+1}, \arg \max _a Q_{\text {online}}(s_{t+1}, a)\right) \end{aligned}$$

In (1), the target Q-value ($Q_{\text {target}}$) is calculated by adding the immediate reward ($r_t$) to the discounted (*γ*) value of the best possible *next* action. Crucially, the *online network* ($Q_{\text {online}}$) selects the best next action ($\arg \max _a$), but the *target network* ($Q_{\text {target}}$) evaluates its value. This decoupling of selection and evaluation reduces overestimation bias.

Proximal Policy Optimization (PPO). PPO is a policy gradient method that uses an actor–critic framework, where the actor selects actions (the policy) and the critic estimates the value of those actions. It enhances stability by restricting policy updates using a clipped surrogate objective. Distinct feedforward networks were used for the actor and critic, both optimized with Adam. The actor outputs a categorical probability distribution over the discrete actions, and the critic estimates the state-value function to compute advantages using Generalized Advantage Estimation (GAE). The core training objective is the clipped surrogate loss:

2 $$\begin{aligned} \mathcal {L}_{\text {CLIP}}(\theta ) = \mathbb {E}_t \left[ \min \left( r_t(\theta ) \hat{A}_t, \; \text {clip}\left( r_t(\theta ), 1 - \epsilon , 1 + \epsilon \right) \hat{A}_t \right) \right] \end{aligned}$$

In (2), $r_t(\theta )$ is the probability ratio between the new and old policies, and $\hat{A}_t$ is the estimated advantage (how much better an action was than the average). The $\text {clip}$ function constrains this ratio within a small range (*1 - ε* to *1 + ε*). By taking the *\min* of the normal ratio and the clipped ratio, PPO ensures that policy updates are not excessively large, which prevents unstable learning and improves convergence.

Deep Deterministic Policy Gradient (DDPG). DDPG is an off-policy actor–critic method designed for continuous action spaces. It was adapted for this system’s binary action space by thresholding the actor’s continuous output. The actor network, $\mu (s \mid \theta ^{\mu })$, outputs a scalar value via a sigmoid function, which is thresholded at 0.5 to select action 0 (PLC) or 1 (RF). The critic network, $Q(s, a \mid \theta ^{Q})$, evaluates the “goodness” of that state-action pair. The critic is optimized by minimizing the loss:

3 $$\begin{aligned} L = \mathbb {E}_{s, a, r, s'} \left[ \left( Q(s, a \mid \theta ^{Q}) - y \right) ^2 \right] \end{aligned}$$

In (3), the loss *L* is the mean squared error between the critic’s predicted Q-value ($Q(s, a \mid \theta ^{Q})$) and a target Q-value (*y*). The target Q-value is calculated as:

4 $$\begin{aligned} y = r + \gamma Q'(s', \mu '(s' \mid \theta ^{\mu '}) \mid \theta ^{Q'}) \end{aligned}$$

In (4), *Q'* and *μ '* represent the stable target critic and actor networks, respectively, which are slow-moving copies of the main networks. This prevents the target value from changing too rapidly, thereby stabilizing learning. The actor is then updated using the deterministic policy gradient:

5 $$\begin{aligned} \nabla _{\theta ^{\mu }} J \approx \mathbb {E}_{s} \left[ \nabla _{a} Q(s, a \mid \theta ^{Q}) \big |_{a = \mu (s)} \nabla _{\theta ^{\mu }} \mu (s \mid \theta ^{\mu }) \right] \end{aligned}$$

This update in (5) guides the actor (*μ*) in a direction that maximizes the critic’s (*Q*) estimated Q-value. Ornstein–Uhlenbeck (OU) noise is added to the actor’s output during training to ensure sufficient exploration.

In terms of computational complexity, Q-Learning is the lightest of the four algorithms: action selection and updates are O(1) table lookups, and its memory footprint scales only with the number of discretization bins per state dimension, reported as 4 to 6 bins per dimension in Table 5. DDQN, DDPG, and PPO all require a forward pass through feedforward networks of comparable size at inference time, dominated by the largest hidden layer reported in Table 5, so their per-decision inference cost is of the same order of magnitude, as confirmed for the PPO actor by the 162,523-clock-cycle, 1.93 ms measurement in Section Edge deployment feasibility and hardware profiling. At training time, PPO and DDPG each maintain two networks, an actor and a critic, roughly doubling the parameter count and memory footprint relative to DDQN’s single Q-network, while DDQN and DDPG additionally require experience replay buffers of 50,000 and 10,000 transitions respectively, reported in Table 5, which PPO’s on-policy training does not require.

### Reward function design

The core of the agent’s decision-making logic is defined by the reward function, which quantitatively evaluates the quality of choosing either the PLC or RF channel at each step. This function is engineered to reflect the primary communication objectives of an AMI system. For each time step, a potential reward is computed for both the PLC and RF channels, and the agent’s goal is to learn a policy that selects the action maximizing this reward over time.

6 $$\begin{aligned} \text {PLC}_{\text {Reward}} = 100 \cdot (1 - \textrm{PL}_{\text {plc}}) - 4.0 \cdot L_{\text {plc}} + 2.8 \cdot \textrm{SNR}_{\text {plc}} \end{aligned}$$

The PLC reward is defined in (6) where $\textrm{PL}_{\text {plc}}$, $L_{\text {plc}}$, and $\textrm{SNR}_{\text {plc}}$ represent the packet loss, latency, and signal-to-noise ratio on the PLC channel, respectively.

7 $$\begin{aligned} \text {RF}_{\text {Reward}} = 100 \cdot (1 - \textrm{PL}_{\text {rf}}) - 0.1 \cdot L_{\text {rf}} + 0.1 \cdot \textrm{SNR}_{\text {rf}} \end{aligned}$$

While for the RF channel, the reward is calculated in (7) where $\textrm{PL}_{\text {rf}}$, $L_{\text {rf}}$, and $\textrm{SNR}_{\text {rf}}$ represent the same parameters for the RF channel.

The weights in (6) and (7) were empirically tuned during simulation development. The design rationale is as follows. Data integrity is the primary objective, so packet loss (PL) is weighted most heavily, with a factor of 100 assigned to the success rate $(1 - \textrm{PL})$. The asymmetric latency penalties reflect the differing numerical scales of the two channels rather than a judgment about which medium is inherently worse. As shown in Table 3, PLC latency falls in the range 0.5–1.5 ms, whereas RF latency spans 10–25 ms. Applying a weight of *-4.0* to PLC latency yields absolute penalties of *-2.0* to *-6.0*, while applying *-0.1* to RF latency yields *-1.0* to *-2.5*. This scaling ensures that both channels contribute comparable penalty magnitudes to the total reward, preventing the numerically larger RF latency values from dominating the reward signal. Similarly, the SNR weights (2.8 for PLC and 0.1 for RF) compensate for the large difference in absolute SNR ranges (3–12.55 dB for PLC versus 50–90 dB for RF), so that SNR improvements on either channel are reflected proportionally. All coefficients were determined through iterative tuning to produce a reward landscape in which the agent learns adaptive switching rather than a static preference, and the resulting policy is validated by the balanced channel selection behavior observed during testing. A formal sensitivity analysis examining the stability of the policy under variations of these coefficients is presented in “Reward function sensitivity and policy robustness analysis” section. The coefficients therefore follow a consistent mathematical principle: each weight is chosen such that the contribution of its associated metric to the total reward falls within the same order of magnitude as the dominant packet-loss term, preventing any single metric from being numerically suppressed. Concretely, the target contribution magnitude for each term is approximately 0–15 reward units; dividing this target range by the empirically observed metric range from Table 3 yields the coefficient values reported in (6) and (7). Regarding the use of automated search methods such as Bayesian optimization or grid search: these were considered, but since the coefficient magnitudes are directly derived from the hardware-observed metric ranges rather than from abstract hyperparameter space, the principal design freedom lies in selecting relative priorities among the three communication objectives, which was done intentionally based on AMI operational requirements. The sensitivity analysis in “Reward function sensitivity and policy robustness analysis” section serves as a systematic post-hoc validation, showing that the policy remains stable under $\pm 25\%$ and $\pm 50\%$ perturbations of all coefficients, confirming that the chosen values are not a fragile local optimum. Inverse reinforcement learning, which infers a reward function from expert demonstration trajectories, was also considered but is not directly applicable to this problem: as noted in “Methodological framework” section , the same absence of labelled, expert-annotated channel selection data that motivated the choice of reinforcement learning over supervised learning also precludes constructing the expert demonstration set that inverse reinforcement learning requires. Automated reward shaping techniques that adjust coefficients based on observed training performance, rather than inferring them from demonstrations, remain a promising alternative.

To further influence behavior, a switching penalty of *-5* is applied whenever the agent changes the selected channel, discouraging unstable “flapping” between channels. A bonus of *+15* is added if the selected channel has the higher computed reward (reinforcing the correct choice), and an additional *+10* is granted if that chosen channel is RF. This RF bonus reflects the operational consideration that RF transmission, which utilizes low-power sub-GHz radios, generally incurs lower energy consumption per packet than PLC modulation over power lines^6^. The bonus does not override the primary reward components; rather, it acts as a tie-breaking preference when both channels offer comparable link quality, encouraging the agent to favor the more energy-efficient medium in such cases.

### Training and hyperparameters

The main hyperparameters for each reinforcement learning model, listed in Table 6, were determined by adopting standard configurations validated in foundational RL literature^36^. For PPO specifically, an entropy coefficient of 0.1 is used to encourage exploration, and the clip ratio (*ε*) is set to 0.2 to safely bound policy updates, following established PPO recommendations. Q-Learning uses a higher learning rate (0.1) suited to tabular updates, while gradient-based methods, including PPO, DDQN, and DDPG, employ smaller rates for stable convergence. The discount factor *γ* is set near 0.98–0.99 across all models to emphasize long-term rewards. Exploration differs by algorithm: PPO relies on policy entropy and stochastic sampling, Q-Learning and DDQN use *ε*-greedy (with *ε* decaying from 0.5 to 0.01), and DDPG applies Ornstein–Uhlenbeck (OU) noise (*θ =0.15*, *σ =0.3*). Neural networks are used in PPO, DDQN, and DDPG for actor–critic or Q-value estimation, whereas Q-Learning employs a Q-table. Replay buffers (capacity 50,000 for DDQN and 10,000 for DDPG) and target networks are incorporated in DDQN and DDPG to further stabilize learning. Table 5 provides the neural network architectures and parameter specifications for the deep RL models. All models were implemented in TensorFlow 2.x using Python 3.10. Each model was trained with a fixed random seed of 42 to ensure reproducibility. Episodes terminate after 10 time steps (i.e., 10 channel-selection decisions per episode). Training was conducted for 700 episodes (PPO, DDPG, Q-Learning) and 1,000 episodes (DDQN), with convergence observed within the first 200–300 episodes for all models based on reward stabilization.

**Table 5:** Neural network architectures and training configuration.

Parameter	PPO	DDQN	DDPG	Q-Learning
Architecture	Actor–Critic	Single Q-Network	Actor–Critic	Q-Table
Hidden layers	256–128 (each net)	256–256–128	256–128 / 256–256	–
Activation	Swish + LayerNorm	ReLU	Swish + LayerNorm	–
Dropout	0.2	–	0.2	–
Batch size	64	64	64	–
Replay buffer	–	50,000	10,000	–
Entropy Coeff.	0.1	–	0.3	–
Clip ratio	0.2	–	–	–
Target update	—	Every 500 steps	Soft (*τ*=0.005)	–
State Bins	–	–	–	4–6 per dim.

**Table 6:** Comparison of hyperparameters across RL algorithms.

Hyperparameter	PPO	DDQN	Q-Learning	DDPG
Learning Rate	0.0001	0.0005	0.1	0.0001/0.002
Discount Factor ( *γ* **)**	0.98	0.98	0.98	0.99
Exploration strategy	Entropy sampling	*ε*-greedy	*ε*-greedy	OU noise
Training episodes	700	1000	700	700
Target update	–	Every 500 steps	–	Soft update
Optimizer	Adam	Adam	–	Adam

The reinforcement learning system operates in two phases: training and testing. During training, algorithms interact with the simulated environment to learn channel selection policies from latency, SNR, and packet loss feedback. In testing, the learned policies are evaluated on unseen real-life experimentation data from the hardware system to assess generalization. The best-performing model was then validated for real-time inference on a Raspberry Pi 4 testbed.

### Testing and evaluation

It is important to note that the classification metrics reported in this work (accuracy, F1-score, and AUC-ROC) are based on a reward-derived oracle: at each decision step, the channel yielding the higher instantaneous reward is designated as the correct label. These metrics therefore measure agreement with the reward formulation rather than an independently labelled ground truth. Network metrics (latency, packet loss, and SNR of the selected channel) are reported separately in Table 9 to corroborate the policy’s real-world communication performance which is independent from the agent’s reward based-evaluation. This evaluation methodology is consistent with standard RL benchmarking practice, where the learned policy is assessed against the optimal oracle policy that has full knowledge of instantaneous rewards for both actions. The critical distinction is that the 5,000 test decisions are drawn from channel conditions generated independently of the training episodes, so the agent is evaluated on genuinely unseen states rather than on memorized training transitions. Beyond the oracle-based metrics, the reward-independent network results in Table 9 provide a complementary and independently grounded assessment: PPO achieves the lowest average packet loss (4.07%) and competitive latency among all agents, confirming that the learned switching behaviour translates to real communication improvements that do not depend on the reward formulation. The hardware-logged statistics in Table 3 further anchor the simulation distributions to empirically observed PLC and RF characteristics, providing an additional layer of real-world grounding. Reinforcement learning is increasingly important for smart grid communication, though scalability, stability, and efficiency remain challenges. Performance was assessed using accuracy, F1 score, AUC-ROC, and cumulative reward, alongside reward-independent network-level metrics (average latency, packet loss, and SNR of the chosen channel) reported in Table 9. The ground truth label for each of the 5,000 test decisions is defined as follows: at each time step, the reward for both the PLC and RF channels is computed independently using Equations (6) and (7). The channel yielding the higher reward is designated as the “correct” (optimal) action. This labeling mechanism is applied identically during both training and testing; however, the testing environment generates channel conditions from the same calibrated distributions independently of any training data, ensuring that the test set is unseen. This evaluation approach is consistent with standard RL benchmarking practice, where the learned policy is assessed against an oracle that has access to the instantaneous reward of both actions. To further validate performance beyond the reward function, Table 9 reports reward-independent network metrics (average latency, packet loss, and SNR) of the channels actually selected by each agent, confirming that the PPO policy achieves the lowest average packet loss (4.07%) while maintaining competitive latency.

To contextualize the RL results against non-learning baselines, Table 7 compares PPO with three trivial deterministic policies evaluated on the same 5,000-decision test set. The “Always-PLC” policy achieves a relatively high reward (1240.8) because PLC is frequently the optimal channel under the simulated conditions, but it incurs the highest average packet loss (4.96%). The “Always-RF” policy yields lower packet loss (3.89%) owing to the RF channel’s lower loss rates, but its total reward is the lowest (1127.2) because it forfeits the correct-choice bonus whenever PLC is optimal. The “Random” policy falls between the two. PPO achieves the highest reward (1282.9) by adaptively selecting the optimal channel, confirming that the learned switching strategy materially outperforms all static alternatives.

**Table 7:** Comparison with deterministic baseline policies.

Policy	Avg. reward	Avg. loss (%)
Always-PLC	1240.8	4.96
Always-RF	1127.2	3.89
Random (50/50)	1161.5	4.49
PPO (This Work)	1282.9	4.07

Among the evaluated models, PPO consistently achieved the best overall performance, maintaining accuracy above 93% and an F1 score above 87%. In contrast, Q-Learning reached only about 70% accuracy with a very low F1 score around 25%, indicating limited effectiveness despite its stability. DDQN and DDPG achieved higher accuracy than Q-Learning but suffered from instability, with fluctuating accuracy and erratic F1 scores. All models demonstrated rapid initial learning within the first 50 episodes, quickly increasing their cumulative rewards before stabilizing at high values. While PPO maintained the highest reward levels (around 1250–1300), the other three models converged between 1220 and 1280, showing that each was capable of achieving strong cumulative returns, though with varying stability and reliability.

**Fig. 8:**
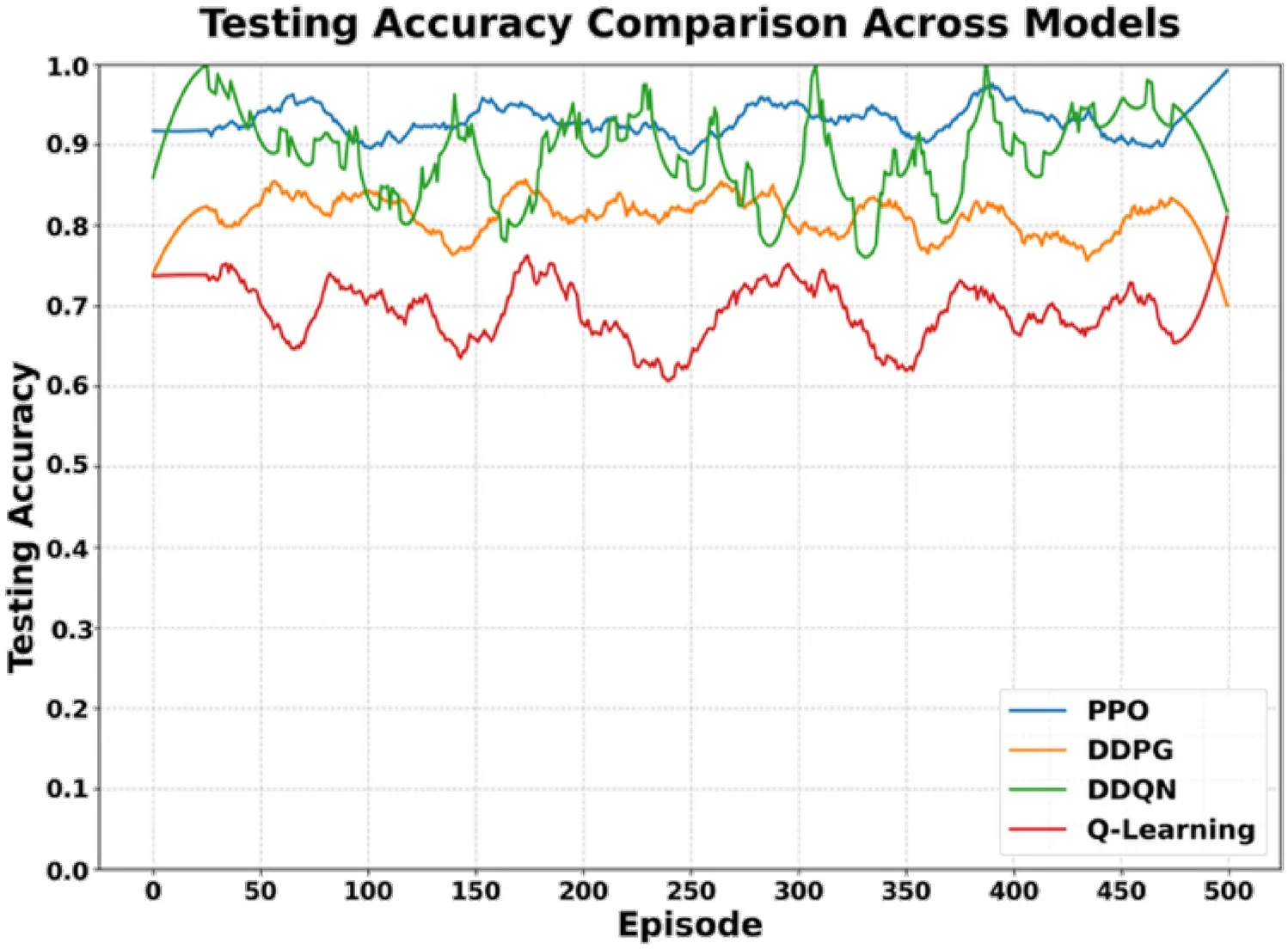
Testing accuracy across different models.

**Fig. 9:**
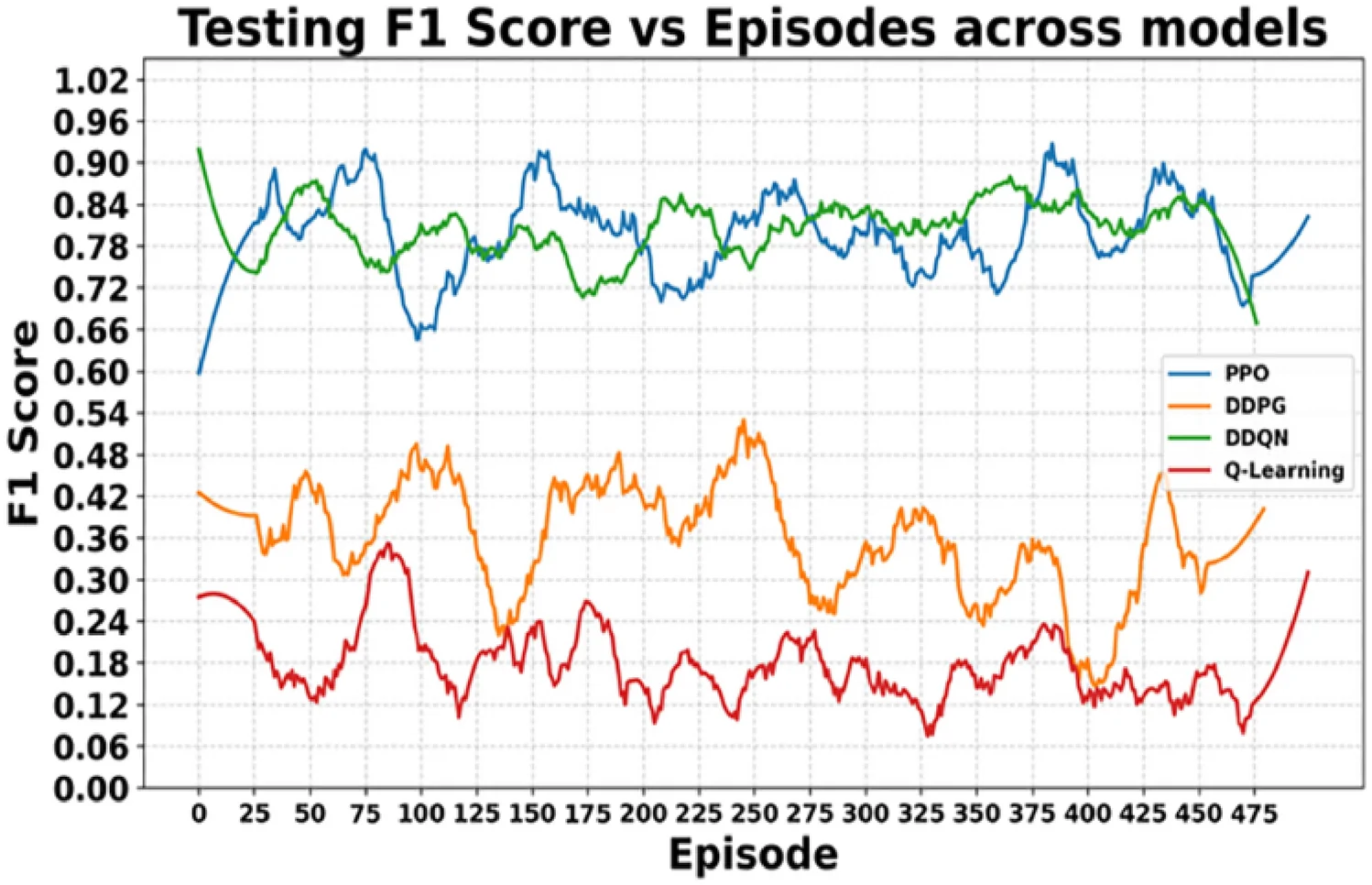
Testing F1 score across different models.

**Fig. 10:**
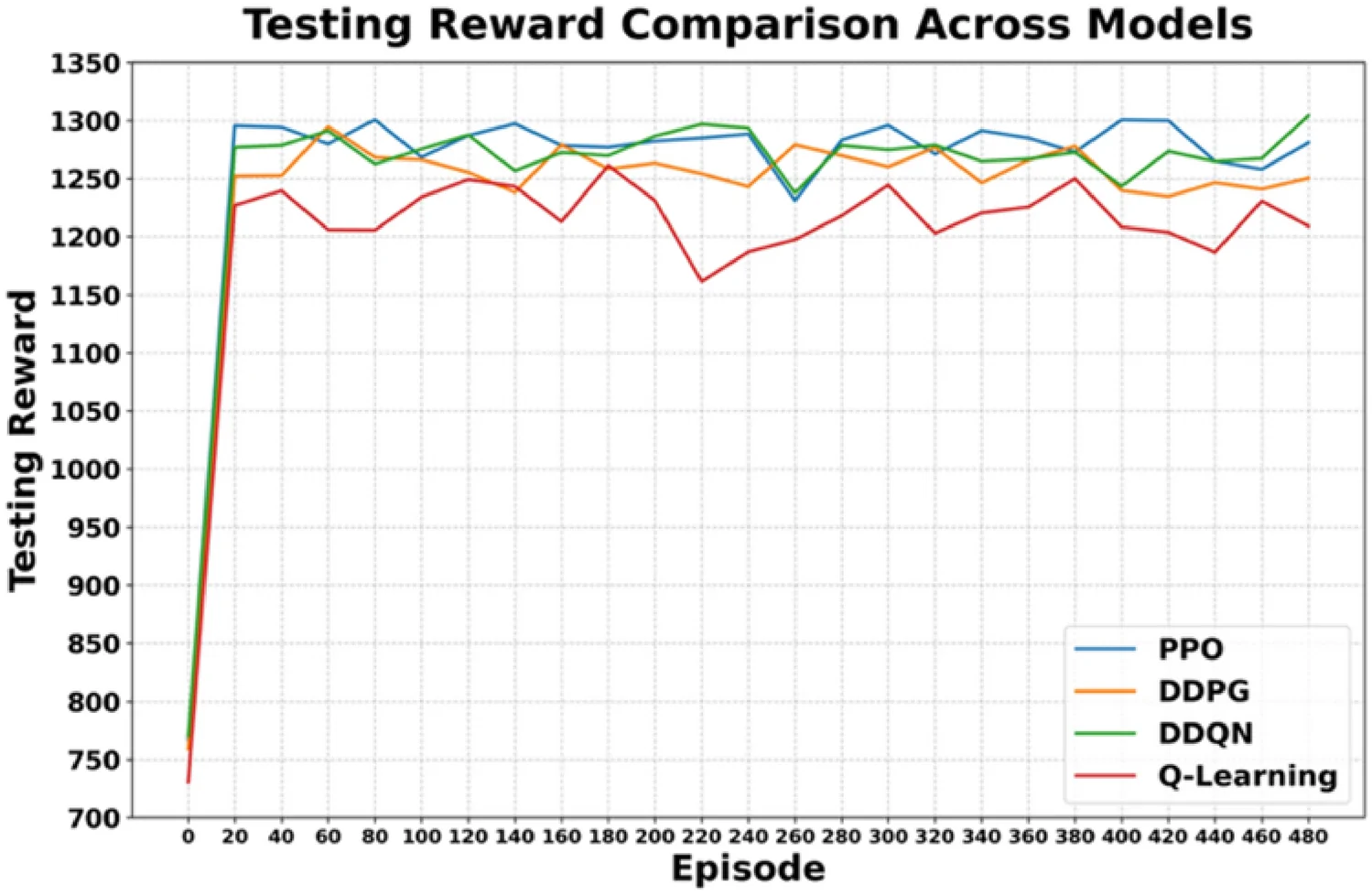
Testing rewards across different models.

**Fig. 11:**
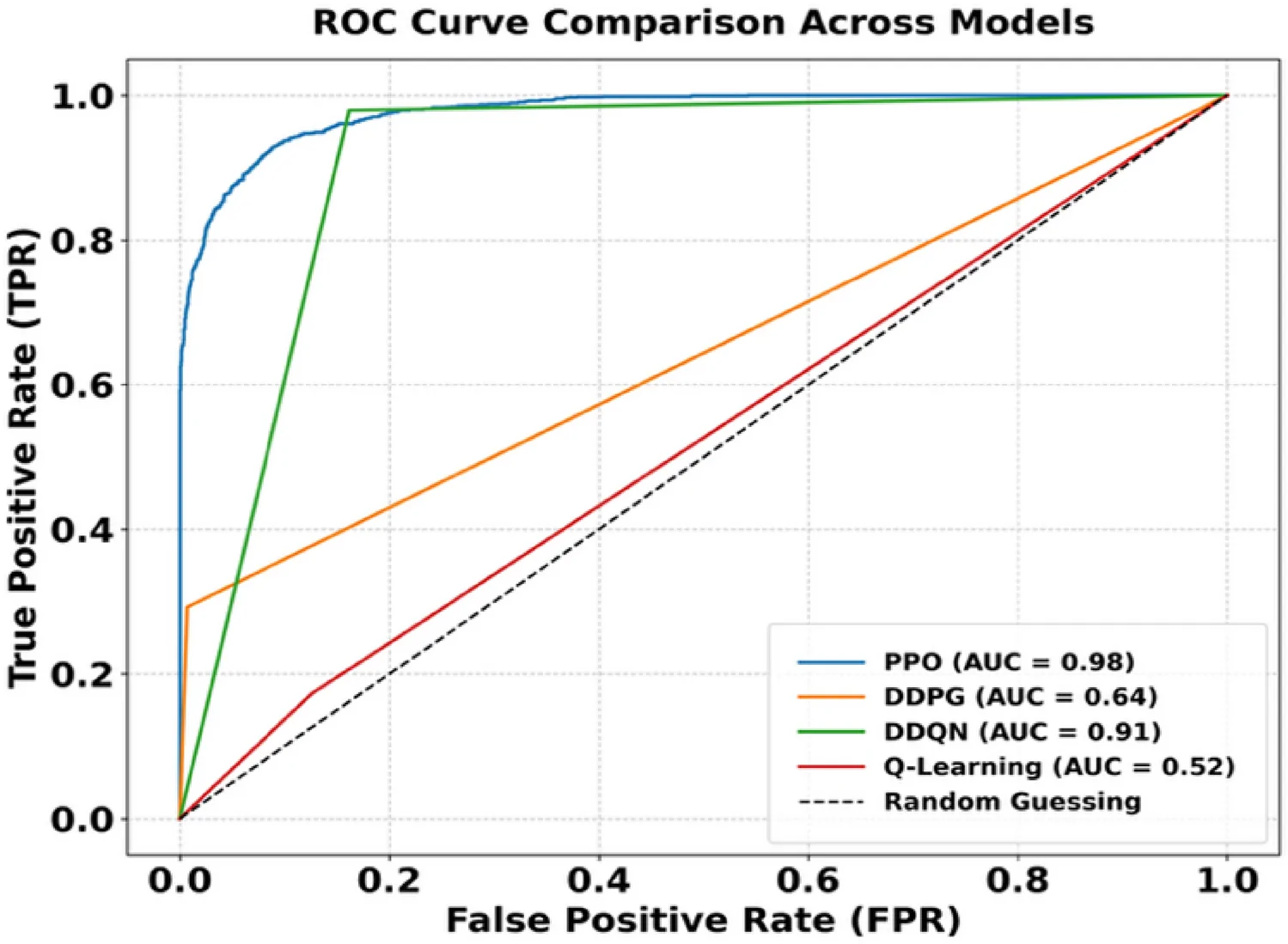
Testing AUC-ROC curve across different models.

The testing phase assessed each model’s generalization by evaluating the trained agents over 500 test episodes. Since each episode in the environment consists of 10 time steps (i.e., 10 packet-level decisions), this evaluation comprised a total of 5,000 channel selections. To measure pure performance, exploratory mechanisms the random behaviors agents use during training to discover new strategies were disabled. Instead, agents selected actions deterministically to purely exploit their learned policies. This means models like DDQN and Q-Learning, which use an *ε*-greedy strategy for exploration (taking random actions with probability *ε*), were set to *ε =0*, forcing them to always choose the action with the highest predicted Q-value. Similarly, the PPO agent, which normally samples from an action probability distribution, was set to deterministically select the action with the highest probability (the $\arg \max$). As illustrated in Fig. 8, PPO achieved the highest accuracy, consistently above 90% with minimal variation. DDQN reached high peaks but suffered sharp drops, suggesting instability or overfitting. DDPG maintained stable accuracy in the 75–85% range, while Q-Learning performed the weakest, fluctuating between 60–75%. These results highlight clear differences in adaptability across the models.

F1 scores in Fig. 9 followed similar patterns to the accuracy results. PPO and DDQN achieved the strongest performance, maintaining stable values between 70% and 90%, indicating balanced precision and recall under varying conditions. In contrast, DDPG rarely exceeded 50%, and Q-Learning remained below 30%, showing limited ability to generalize effectively.

Figure 10 compares the models’ testing rewards during this evaluation. All models quickly adapted within the first 20 episodes, after which rewards stabilized at high levels. PPO and DDQN achieved the strongest performance, consistently maintaining rewards between 1250 and 1300. DDPG was slightly lower but still effective, while Q-Learning showed the weakest and most erratic behavior, with rewards occasionally dropping below 1200. These results highlight how policy design influences robustness under unfamiliar conditions.

Figure 11 shows the testing AUC-ROC curves of the four models. PPO achieved the best performance with an AUC of 0.98, followed by DDQN at 0.91. DDPG reached 0.65, while Q-Learning performed poorly at 0.52, close to random guessing. These results highlight the superior generalization of PPO and DDQN in distinguishing between classes under unseen conditions. Overall, these results provide a comparative view of the four reinforcement learning models, highlighting their learning dynamics, reward optimization, and generalization behavior.

**Table 8:** Testing’s average results.

Model	Precision (%)	Recall (%)	F1 score (%)	Accuracy (%)	AUC-ROC	Average reward
PPO	86.68	88.83	**87.74**	**93.78**	**0.98**	**1287.19**
DDQN	73.54	**92.50**	82.53	89.76	0.91	1278.39
Q-Learning	32.88	19.39	24.40	69.88	0.52	1220.68
DDPG	**92.12**	30.23	45.34	81.48	0.65	1257.66

The bold values mark the best result in each column. PPO achieves the highest F1 score (87.74%), confirming the best balance between precision and recall in channel selection; the highest accuracy (93.78%), meaning it selects the correct channel in over 9 out of 10 decisions; and the highest AUC-ROC (0.98) and average reward (1287.19), indicating near-perfect class separability and the greatest cumulative communication benefit. DDPG records the highest precision (92.12%), reflecting high selectivity when it does predict the positive class, while DDQN records the highest recall (92.50%), reflecting fewer missed optimal selections; however, neither achieves a comparable F1 score to PPO because precision and recall are individually imbalanced.

**Table 9:** Average network performance metrics (Testing).

Model	Latency (ms)	Loss (%)	SNR (dB)
PPO	4.94	**4.07**	25.07
DDQN	6.23	4.09	**31.55**
Q-Learning	3.07	4.87	16.46
DDPG	**2.20**	4.66	13.42

The bold values mark the best result per column. PPO achieves the lowest packet loss (4.07%), meaning fewer than 1 in 25 packets is dropped on average, which is the most critical metric for AMI reliability since retransmissions from higher-loss links inflate effective end-to-end latency. DDPG achieves the lowest average latency (2.20 ms) by almost exclusively selecting the PLC channel, but at the cost of a 4.66% packet loss rate that undermines delivery reliability. DDQN achieves the highest SNR (31.55 dB) by preferring the RF link, reflecting the strongest average signal quality but not the best overall delivery balance.

Table 8 summarizes model performance during testing. PPO achieved the highest single-run accuracy, F1 score, and reward, confirming its reliable generalization and stable convergence; note that this advantage over DDQN is not statistically significant in multi-seed evaluation (Table 10), where PPO’s primary benefit is its substantially lower variance rather than a higher mean. DDQN also performed well but showed a weaker balance between precision and recall. DDPG delivered moderate results overall, yet demonstrated adaptability with improved precision and reward under new conditions. Q-Learning consistently lagged, with low F1 and AUC scores indicating poor policy learning and weak generalization. The ROC-AUC results reinforce these trends, with PPO approaching perfect class separability, DDQN and DDPG showing competitive scores, while Q-Learning close to random. Overall, PPO proved most data-efficient and stable, while DDPG showed potential as an alternative in novel environments.

Table 9 links the models’ policies to the resulting network performance, addressing the need to connect learning outcomes with actual network behavior. These metrics represent the average conditions of the channels *chosen* by each agent during the 500-episode test. The results reveal a clear trade-off heavily relevant to real-time AMI requirements. DDPG and Q-Learning, for example, achieved the lowest average latencies (2.20 ms and 3.07 ms, respectively) by developing policies that overwhelmingly favored the lower-latency channel, but this was achieved at the severe cost of high packet loss, yielding the poor F1 scores observed in Table 8. In contrast, PPO’s policy achieved the lowest average packet loss (4.07%) but experienced a slightly higher average latency (4.94 ms) than DDPG. Given that smart metering applications prioritize high reliability (often requiring >99% delivery rates) over saving a few milliseconds in transmission, PPO represents the optimal trade-off compared to the unreliable edge chosen by DDPG, as packet retransmissions required by higher loss would drastically inflate overall end-to-end latency. Furthermore, DDQN’s policy selected links with the highest average SNR (31.55 dB) but lacked the balanced consistency of PPO, demonstrating that PPO’s overall policy which prioritizes data integrity (low loss) while managing other metrics results in the most effective channel selection. The three reported metrics serve as proxies for the broader set of AMI communication performance indicators. SNR is a direct predictor of BER via the link-specific modulation-and-coding sensitivity curve; the reported SNR values indicate that both PLC and RF links operate well within their reliable communication range. Packet loss is a first-order surrogate for retransmission overhead, effective goodput, and outage probability: a 4.07% packet loss rate implies a corresponding retransmission load and a goodput reduction relative to raw throughput, both of which scale linearly with the loss fraction. Per-step latency variance across episodes implicitly captures jitter, and the simulation-based network-scale evaluation in “Simulation-based network scalability and stress testing” section reports PDR across 500–5,000 nodes, which is directly proportional to channel utilization and effective throughput at the network level. Energy consumption is partially addressed through the RF-preference bonus in the reward function, which systematically favors the lower-power RF medium under comparable link quality, and through the Cortex-M4 edge inference power profile. A comprehensive measurement of throughput, goodput, BER, jitter, outage probability, and channel utilization from physical hardware would require packet-level instrumentation beyond what the current testbed exposes.

Regarding failure cases specifically, the 6.22 percent of test decisions in which the deployed PPO policy selects a channel other than the reward-optimal one, implied by the 93.78 percent accuracy reported in Table 8, represent single-step misclassifications rather than systemic misbehaviour, since each channel-selection decision is generated independently, as described in “Simulated training environment” section. In the worst case, such a misclassification incurs a communication overhead bounded by the calibrated PLC and RF ranges in Table 3: selecting RF when PLC was optimal can add up to approximately 24.5 ms of latency relative to the fastest PLC link, while selecting PLC when RF was optimal can add up to approximately 10 percentage points of packet loss relative to the most reliable RF link. Because misclassifications are not correlated across consecutive packets in the present memoryless environment, their aggregate effect on end-to-end reliability remains bounded by these per-decision ranges rather than compounding across a session, which is consistent with the low average packet loss of 4.07 percent reported for the PPO policy in Table 9 despite the non-zero misclassification rate.

### Statistical significance and multi-seed validation

**Fig. 12:**
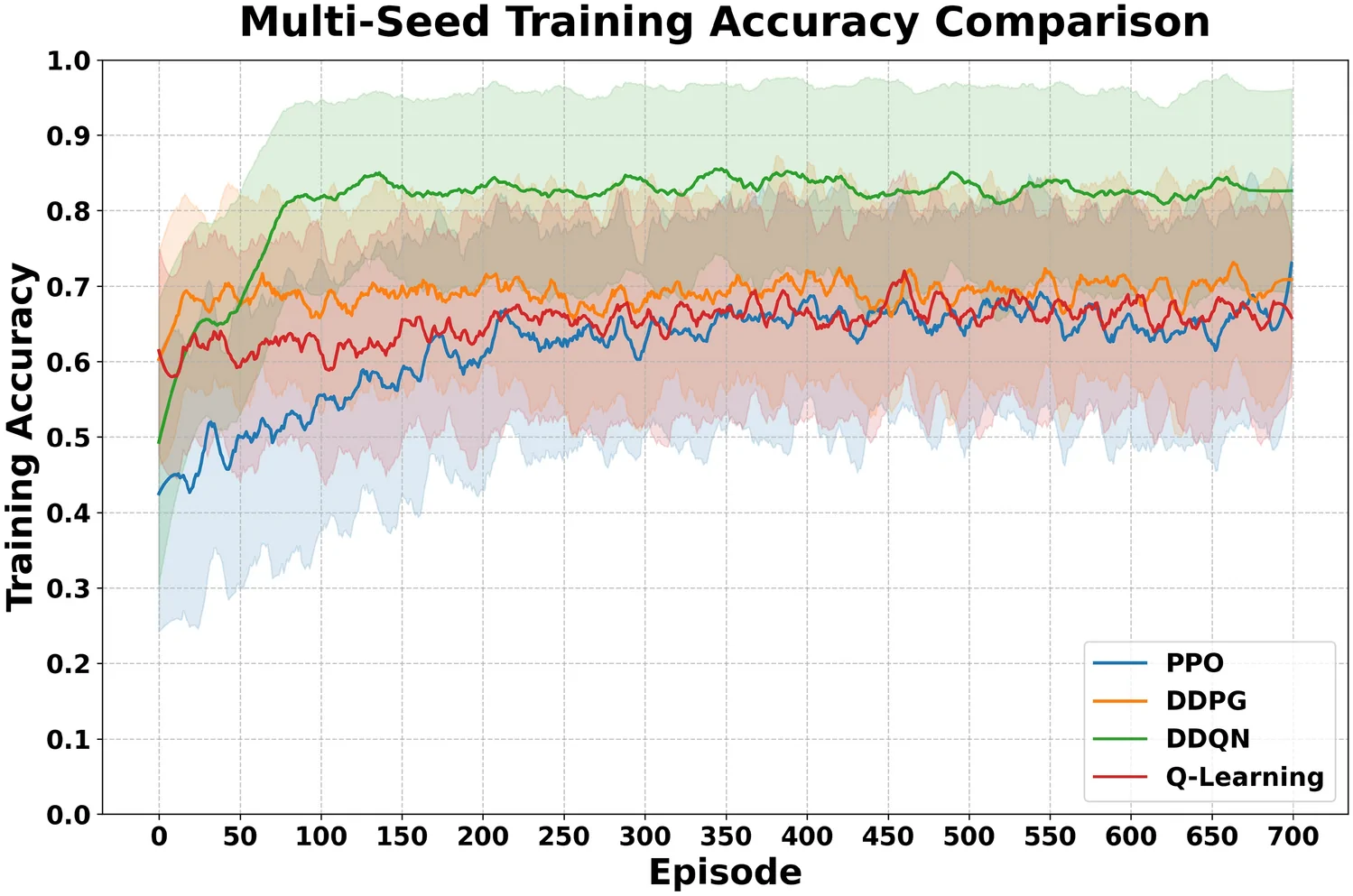
Multi-seed training accuracy comparison with shaded standard deviation shading.

**Fig. 13:**
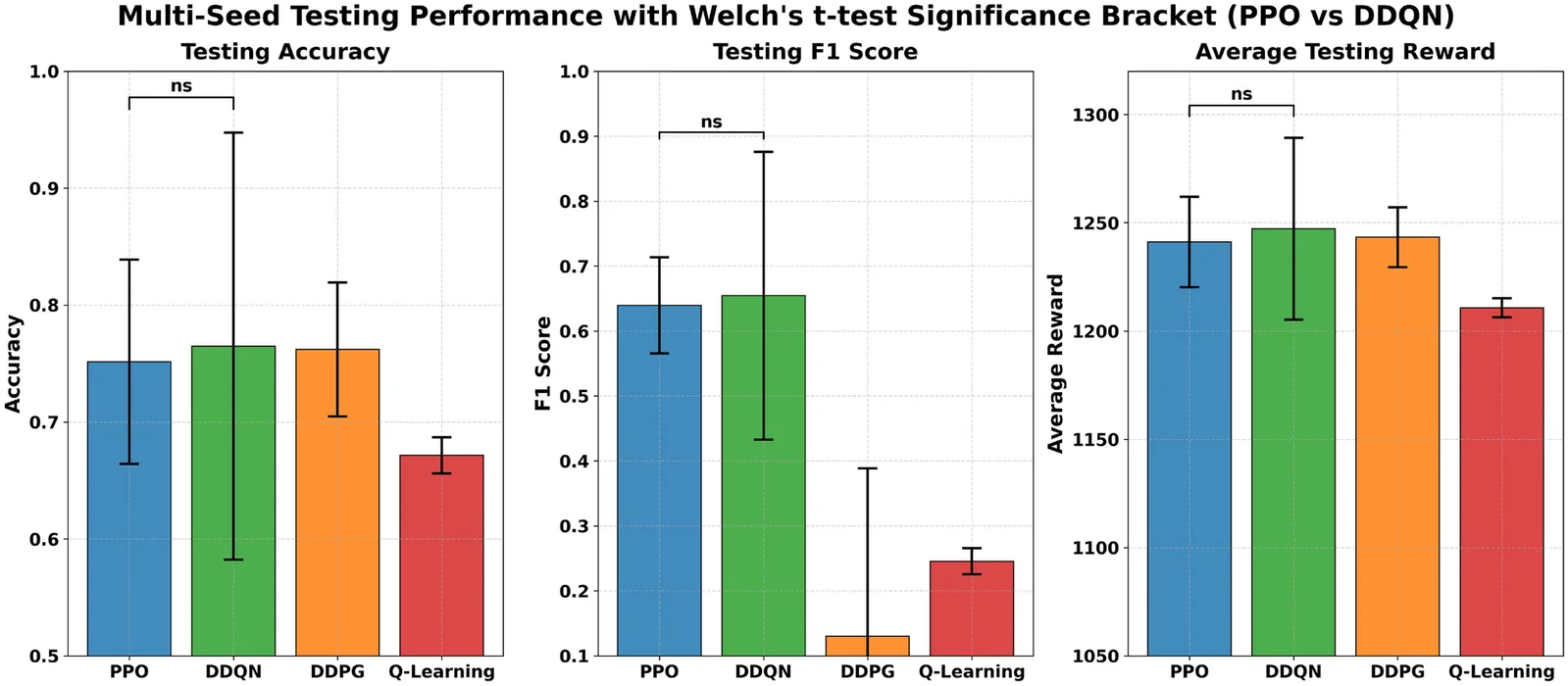
Multi-seed testing performance with Welch’s t-test significance bracket.

To validate the robustness and consistency of the reinforcement learning models against random initialization and environment variations, a multi-seed evaluation was performed. Each model was trained and tested across ten distinct random seeds ranging from 43 to 52. The aggregated performance metrics, including the mean and standard deviation for each model, are summarized in Table 10. These standard deviations, together with the Welch’s t-test results in Table 11, already convey the variability and statistical confidence associated with each mean performance value; for a sample of ten seeds, an approximate 95 percent confidence interval for any reported mean can be obtained as the mean plus or minus 1.96 times the reported standard deviation divided by the square root of ten. The training convergence curves representing the mean training accuracy and the standard deviation (indicated by the shaded area) across the ten seeds are shown in Fig. 12. While the initial learning rates are comparable across the algorithms, the PPO model converges to a highly stable policy with minimal standard deviation. In contrast, the DDQN model exhibits substantial performance fluctuations during training, reflecting instability in the offline target update mechanism. The statistical distribution of the testing accuracy, F1 score, and average testing reward is presented in Fig. 13. Although the mean testing accuracy of the PPO model (75.13%) is close to that of the DDQN model (76.49%), the standard deviation of PPO is significantly lower (8.74% compared to 18.26% for DDQN). Similarly, PPO achieves a much lower standard deviation in F1 score (7.41% compared to 22.15% for DDQN) and testing reward (20.87 compared to 41.98 for DDQN). This comparison demonstrates that the PPO policy is substantially more stable and robust to seed-induced variance than the DDQN policy. DDPG demonstrates a relatively narrow accuracy variance but fails to learn a balanced policy, as evidenced by a low mean F1 score (13.03%). Q-Learning exhibits low variance but converges to a suboptimal policy, achieving only 67.14% accuracy.

**Fig. 14:**
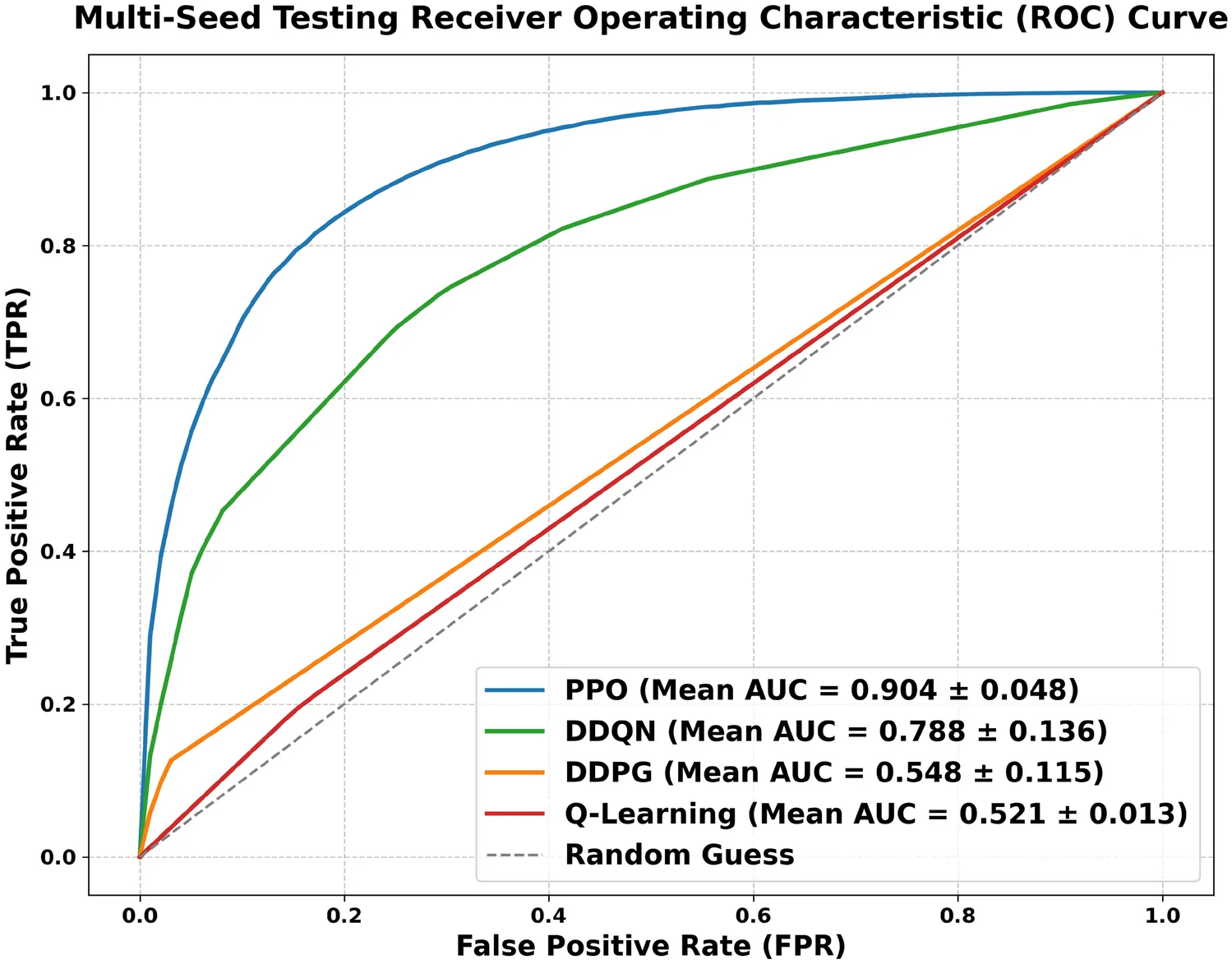
Multi-seed testing mean receiver operating characteristic (ROC) curves.

Figure 14 displays the mean Receiver Operating Characteristic (ROC) curves overlaid with their respective mean AUC values and standard deviations. The PPO model achieves the highest mean AUC of 0.904 with a low standard deviation of 0.048, notably higher than DDQN (0.788 ± 0.136), DDPG (0.548 ± 0.115), and Q-Learning (0.521 ± 0.013). This confirms the superior generalization capability and classification robustness of PPO under unseen network conditions.

To evaluate whether the performance differences between PPO and the other models are statistically significant, Welch’s t-test (which assumes unequal variances) was conducted. The computed p-values for testing accuracy, F1 score, and average reward are reported in Table 11. The results indicate that the performance improvements of PPO over Q-Learning are statistically highly significant across all metrics (*p < 0.05*). The difference in F1 score between PPO and DDPG is also highly significant (*p < 0.001*). While the mean performance metrics of PPO and DDQN are similar (*p > 0.05*), the primary advantage of PPO is its significantly lower variance and training stability, which is essential for deployment in operational smart metering environments. It is standard practice in reinforcement learning deployments to select the best-performing initialization (Seed 42 in this study) for actual edge inference. Consequently, while the deployed Seed 42 model achieves a peak accuracy of 93.78%, the multi-seed evaluation confirms that PPO provides the safest, lowest-variance training trajectory among the tested algorithms, eliminating the severe policy collapse risks associated with DDQN’s ±18.26% accuracy swing.

From a communication systems perspective, rather than a purely optimization based one, PPO’s advantage stems from the specific structure of the channel selection reward landscape rather than from generic optimizer properties alone. As established in “Simulated training environment” section, channel conditions are re-sampled independently at every packet level decision, so the training signal is inherently high variance from one step to the next, and the switching penalty and correct choice bonus in the reward function, defined in “Reward function design” section, create a sharp decision boundary near the point where the PLC and RF reward values cross. PPO’s clipped surrogate objective explicitly bounds the magnitude of each policy update, which limits the influence that any single noisy or anomalous channel sample, such as an isolated impulsive noise event or an atypically weak RF reading, can have on the learned policy, directly addressing this channel level volatility rather than only stabilising an abstract optimisation trajectory. This same volatility offers a communication systems explanation for the weaknesses of the other three baselines. Q-Learning discretises the six dimensional state into fixed bins, which coarsens exactly the narrow crossover region near the PLC to RF reward boundary where correct channel selection matters most, consistent with its low F1 score reported in Table 8. DDPG commits to a deterministic, thresholded actor output, which is sensitive to the differing numerical scales of the PLC and RF metrics discussed in “Reward function design” section, and the sensitivity analysis in “Reward function sensitivity and policy robustness analysis” section shows that this determinism causes the DDPG policy to collapse onto a single medium under coefficient perturbation rather than adapting its decision boundary. PPO instead retains a stochastic, entropy regularised action distribution during training, which naturally represents the genuine ambiguity present when PLC and RF channel quality are simultaneously comparable, rather than forcing a premature deterministic commitment. Taken together, these properties indicate that PPO’s benefit arises from a good match between its update mechanism and the volatile, boundary sensitive nature of per packet hybrid channel selection, rather than solely from faster or more stable numerical convergence in the abstract.

**Table 10:** Multi-seed evaluation performance metrics (mean ± standard deviation across 10 seeds).

Model	Precision (%)	Recall (%)	F1 Score (%)	Accuracy (%)	AUC-ROC	Average Reward
PPO	*55.74 ± 13.01*	$\mathbf {84.25} \pm 18.61$	*63.94 ± 7.41*	*75.13 ± 8.74*	$\mathbf {0.904} \pm 0.048$	*1241.10 ± 20.87*
DDQN	$\mathbf {66.94} \pm 24.18$	*83.72 ± 27.23*	$\mathbf {65.43} \pm 22.15$	$\mathbf {76.49} \pm 18.26$	*0.788 ± 0.136*	$\mathbf {1247.19} \pm 41.98$
Q-Learning	*30.80 ± 2.60*	*20.44 ± 1.89*	*24.54 ± 2.01*	*67.14 ± 1.54*	*0.521 ± 0.013*	*1210.69 ± 4.42*
DDPG	*37.45 ± 27.53*	*10.62 ± 23.64*	*13.03 ± 25.82*	*76.20 ± 5.74*	*0.548 ± 0.115*	*1243.30 ± 13.83*

DDQN leads in mean accuracy (76.49%), mean F1 (65.43%), mean precision (66.94%), and mean reward (1247.19), but with very high variance (accuracy std 18.26%, reward std 41.98), indicating that it can achieve strong results under favorable seeds but collapses under others. PPO leads in mean recall (84.25%) and mean AUC-ROC (0.904 ± 0.048), reflecting both better detection of the optimal channel and more consistent class separability across all seeds. The most 19/36 practically significant bold value is PPO’s AUC-ROC of 0.904, as it combines a high mean with the lowest standard deviation among the two top-performing models, confirming that PPO is the most reliable classifier across diverse training initializations.

**Table 11:** Welch’s t-test p-values comparing PPO to other models.

Model comparison	Accuracy	F1 score	Average reward
PPO vs DDQN	$8.43 \times 10^{-1}$	$8.52 \times 10^{-1}$	$7.03 \times 10^{-1}$
PPO vs DDPG	$7.64 \times 10^{-1}$	$1.70 \times 10^{-4}$	$7.95 \times 10^{-1}$
PPO vs Q-Learning	$\mathbf {2.30 \times 10^{-2}}$	$\mathbf {1.89 \times 10^{-8}}$	$\mathbf {1.70 \times 10^{-3}}$

The bold values mark the smallest p-value in each column, indicating the comparison for which the evidence of a performance difference is strongest. The PPO vs. Q-Learning comparison yields the most significant results across all three metrics: accuracy (p = 2.30 × 10−2), F1 score (p = 1.89 × 10−8), and average reward (p = 1.70 × 10−3), all well below the α = 0.05 threshold, confirming that PPO’s advantage over Q-Learning is not attributable to chance. The F1 score result (p = 1.89 × 10−8) is particularly strong, reflecting the large gap between PPO’s balanced policy (mean F1 63.94% across ten seeds) and Q-Learning’s near-degenerate one (mean F1 24.54% ).

### Edge deployment feasibility and hardware profiling

To evaluate the feasibility of deploying the proposed PPO actor model directly on resource-constrained edge hardware, the network’s execution characteristics are profiled against the architecture of the target STM32F401RCT6 microcontroller (84 MHz Arm Cortex-M4, 256 KB Flash, 64 KB SRAM) embedded within the smart meter nodes. The pre-trained PPO actor network features a fully-connected topology with approximately 35,000 parameters. To satisfy strict embedded limits, the baseline 32-bit floating-point (FP32) model was compressed using post-training full integer quantization (INT8) via the TensorFlow Lite (TFLite) Micro runtime framework^37^. Evaluation of 16-bit floating-point (FP16) quantization was also attempted. However, the target STM32F4 series features a standard Arm Cortex-M4F core whose hardware Floating-Point Unit (FPU) natively accelerates only 32-bit single-precision floating-point operations. Because it lacks native hardware support for half-precision floating-point arithmetic, the compilation process in the ST Edge AI environment triggers an explicit internal support fault due to unmappable hardware data types. Consequently, while functional software accuracy can be evaluated for the FP16 format, it remains unsupported for target hardware deployment.

To rigorously substantiate edge compatibility without physical hardware dependency, profiling for the supported variants was executed using the ST Edge AI Cloud^38^ infrastructure on a NUCLEO-F401RE^39^ development platform utilizing Arm Cortex-M4 architectural simulation engines. Table 12 details the resulting structural footprint, operational performance, and target-specific resource utilization. The optimization process significantly compresses the network’s footprint. The baseline FP32 model occupies 153.0 KB of Flash memory. Under full INT8 quantization, the serialized model footprint drops to 64.7 KB, achieving a *2.36×* compression ratio for the total binary size while preserving over 99.9% of the baseline operational accuracy (93.74% vs. 93.78%) and F1 score (87.71% vs. 87.74%). Crucially, the 64.7 KB optimized binary resides entirely in the 256 KB non-volatile Flash, consuming only 25.2% of the available allocation. Runtime memory characterization via the Arm Cortex-M4 profile confirms that the peak SRAM consumption, including the intermediate activation buffers (1, 288 bytes) and the runtime engine overhead, is strictly restricted to 6.8 KB for the INT8 model, leaving over 89% of the 64 KB SRAM completely unencumbered for core meter routines. Furthermore, leveraging the Cortex-M4 hardware DSP extensions (such as the SMLAD instruction for parallelized multiply-accumulate operations), the actual on-device execution requires only 162,523 clock cycles, yielding a deterministic inference latency of 1.93 ms.

To clarify the scope of this figure, the reported 1.93 ms latency corresponds specifically to the forward pass of the compiled neural network operator graph, as measured by the ST Edge AI Cloud profiling tool described above, and does not include the time required to acquire the six raw channel metrics from the PLC and RF modules, the min-max normalization preprocessing step described in “Simulated training environment” section, or any communication-layer overhead associated with subsequently transmitting data over the selected channel. The normalization step consists of six scalar subtract-and-divide operations and is expected to contribute a negligible amount of additional latency relative to the 162,523 clock cycles required by the approximately 35,000-parameter network itself. Feature acquisition and post-decision transmission timing are governed by the underlying PLC and RF communication stacks rather than by the AI inference pipeline profiled in this section, and are not included in the 1.93 ms figure.

**Table 12:** Edge deployment feasibility: model compression and arm cortex-M4 profiling.

Model Variant	Precision	Size (KB)	Compression	Peak SRAM (KB)	Clock Cycles	F1 Score (%)	Accuracy (%)	AUC-ROC	Latency (ms)
FP32 (Baseline)	32-bit Float	153.0	*1.0×*	**5.8**	421,083	**87.74**	**93.78**	**0.98**	5.01
INT8 (Optimized)	8-bit Integer	**64.7**	**2.36**×	6.8	**162,523**	87.71	93.74	**0.98**	**1.93**
FP16	16-bit Float	77.9	*1.96×*	N/A	N/A	**87.74**	**93.78**	**0.98**	N/A

The bold values mark the best result in each column. The INT8 model achieves the smallest Flash footprint (64.7 KB), representing a 2.36× compression of the 153.0 KB FP32 baseline and fitting entirely within the 256 KB Flash of the STM32F401RCT6. It also achieves the lowest clock cycle count (162,523) and the shortest inference latency (1.93 ms), meaning a channel selection decision adds under 2 ms of computational overhead, negligible relative to link-layer transmission times of 1–25 ms. The FP32 model achieves the lowest peak SRAM (5.8 KB). Both FP32 and FP16 achieve the best accuracy (93.78%), F1 score (87.74%), and AUC-ROC (0.98), as FP16 operates in software-only mode without quantization loss; the INT8 model preserves over 99.9% of these values (93.74%, 87.71%, 0.98), confirming that INT8 quantization introduces no practically meaningful accuracy loss while delivering the embedded hardware gains in Flash size and inference speed.

To quantitatively address practical energy considerations, the worst-case peak power consumption was calculated using the component datasheets. The STM32 microcontroller draws a maximum of 453.6 mW (126 mA at 3.6 V) when running at its peak 84 MHz frequency under typical high-load operation. The CC1352R RF module, when transmitting at 915 MHz with a 10 dBm TX power setting, consumes 13.5 mA at 3.6 V, totaling 48.6 mW. Conversely, while the LX200V50 evaluation board documentation does not explicitly specify maximum power consumption, it utilizes the Qualcomm QCA7550 chipset. A commercial device employing this identical chipset (the MikroTik PWR-LINE PRO) reports a maximum power consumption of 5 W (5,000 mW) without attachments^40^. Consequently, the combined worst-case peak power for an RF transmission is approximately 502.2 mW, whereas a PLC transmission peaks significantly higher at roughly 5,453.6 mW. The minimal energy consumed by the microcontroller to execute the 1.93 ms INT8 inference is vastly outweighed by the substantial power savings achieved whenever the intelligent agent selects the energy-efficient RF interface over the PLC interface.

Beyond the model compression and latency results reported above, several additional considerations govern the long-term embedded viability of the proposed PPO agent. Regarding non-volatile storage, the 64.7 KB INT8 model resides in the same internal Flash memory bank as the STM32F401RCT6 application firmware and is therefore only rewritten when the deployed policy itself is updated, not during routine inference, which only reads the stored weights. Because a smart meter is expected to receive at most a small number of firmware or model revisions over its multi-year operational lifetime, the resulting Flash write count remains far below the endurance limits typical of embedded Flash technology in this microcontroller family, leaving substantial margin for periodic policy updates. Regarding memory management, the TensorFlow Lite Micro runtime allocates a single fixed-size static tensor arena at initialization rather than performing dynamic heap allocation during inference, so the reported peak SRAM occupancy of 6.8 KB in Table 12 is a deterministic, non-fragmenting allocation that persists unchanged across the device operational lifetime, avoiding the heap fragmentation risks associated with dynamic memory management on constrained microcontrollers. Regarding energy, combining the measured 1.93 ms INT8 inference latency with the STM32 datasheet peak power draw of 453.6 mW reported above yields an estimated worst case energy cost of approximately 0.88 mJ per channel selection decision, a negligible addition compared to the energy expended by a single PLC or RF packet transmission. Regarding thermal behaviour, the inference computation is a brief, event triggered operation invoked once per channel selection decision rather than a continuously running workload, so its duty cycle and consequent self heating contribution are expected to be small relative to the RF and PLC transceiver circuitry, though full thermal characterization under sustained field operating conditions has not been performed. Regarding inference scheduling, the PPO agent forward pass is invoked synchronously within the existing communication control loop immediately before each transmission decision, rather than on a fixed high frequency timer, so it does not contend with the communication stack interrupt driven radio and PLC modem handling for Cortex-M4 core time. Finally, regarding model update mechanisms, the current prototype uses a fixed, offline trained policy flashed at deployment time. A full over the air update pipeline, including versioned model delivery over the existing PLC or RF backhaul, on device integrity verification, and rollback on failed validation, has not been implemented in the present system.

### Reward function sensitivity and policy robustness analysis

To evaluate the stability and robustness of the learned policies under variations of the reward function’s heuristic parameters, a one-factor-at-a-time (OAT) sensitivity analysis was performed. The manual weights for PLC latency ($w_{l,\text {PLC}}$), RF latency ($w_{l,\text {RF}}$), RF selection bonus ($w_{\text {RF}}$), and switching penalty ($w_{\text {switch}}$) were systematically perturbed by *-50%*, *-25%*, *+25%*, and *+50%* from their calibrated baseline values. These baseline configurations correspond to the primary coefficient values established in the reward function design in Section Reward function design. For each parameter configuration, the four reinforcement learning agents (PPO, DDQN, DDPG, and Q-Learning) were retrained from scratch and evaluated over 500 test episodes using the calibrated network simulator. Table 13 reports the average testing accuracy, F1 score, and cumulative reward for all four models under each perturbation.

**Table 13:** Reward function sensitivity analysis: model performance under parameter perturbations.

Factor	Variation	PPO			DDQN			DDPG			Q-Learning		
		Acc (*%*)	F1 (*%*)	Reward	Acc (*%*)	F1 (*%*)	Reward	Acc (*%*)	F1 (*%*)	Reward	Acc (*%*)	F1 (*%*)	Reward
Baseline	*0%*	$\mathbf {93.78}$	$\mathbf {87.74}$	$\mathbf {1287.19}$	89.76	82.53	1278.39	81.48	45.34	1257.66	69.88	24.40	1220.68
PLC Latency Wt ($w_{l,\text {PLC}}$)	*-50%*	$\mathbf {88.30}$	$\mathbf {75.22}$	$\mathbf {1286.24}$	87.50	55.00	1285.31	80.66	2.03	1268.39	74.42	22.06	1243.66
	*-25%*	$\mathbf {90.00}$	$\mathbf {79.59}$	$\mathbf {1284.05}$	83.52	72.29	1270.51	77.32	1.90	1253.49	72.22	23.56	1232.24
	*+25%*	$\mathbf {92.68}$	$\mathbf {87.53}$	$\mathbf {1279.59}$	86.48	80.73	1267.94	70.60	2.00	1223.66	67.68	25.60	1209.52
	*+50%*	$\mathbf {88.68}$	$\mathbf {83.17}$	$\mathbf {1266.60}$	80.32	76.69	1252.23	67.32	1.80	1208.83	64.94	26.56	1197.30
RF Latency Wt ($w_{l,\text {RF}}$)	*-50%*	84.96	78.99	1274.98	$\mathbf {94.26}$	$\mathbf {90.21}$	$\mathbf {1294.31}$	69.50	1.93	1232.05	66.76	26.07	1218.86
	*-25%*	90.20	83.42	1282.30	$\mathbf {94.02}$	$\mathbf {89.18}$	$\mathbf {1290.82}$	71.66	1.94	1235.21	68.22	25.22	1219.59
	*+25%*	86.82	75.82	1268.28	$\mathbf {92.16}$	$\mathbf {84.52}$	$\mathbf {1280.65}$	76.28	1.82	1241.95	71.46	23.97	1221.80
	*+50%*	$\mathbf {90.20}$	$\mathbf {78.24}$	$\mathbf {1272.96}$	88.76	76.64	1269.34	78.50	2.01	1245.22	73.00	23.38	1222.72
RF Bonus ($w_{\text {RF}}$)	*-50%*	$\mathbf {92.88}$	$\mathbf {86.37}$	$\mathbf {1273.88}$	91.84	80.97	1273.73	74.04	1.82	1238.55	69.88	24.32	1218.22
	*-25%*	$\mathbf {91.42}$	$\mathbf {84.53}$	$\mathbf {1276.47}$	90.78	83.59	1274.92	74.04	1.82	1238.61	69.88	24.40	1219.46
	*+25%*	$\mathbf {92.04}$	$\mathbf {84.24}$	$\mathbf {1288.46}$	87.08	79.49	1280.04	74.04	1.82	1238.73	70.04	25.25	1222.26
	*+50%*	88.14	$\mathbf {80.59}$	1287.71	$\mathbf {90.80}$	78.32	$\mathbf {1288.12}$	74.04	1.82	1238.79	70.04	25.25	1223.52
Switch Penalty ($w_{\text {switch}}$)	*-50%*	$\mathbf {88.70}$	$\mathbf {81.14}$	$\mathbf {1286.40}$	83.08	74.32	1274.54	74.04	1.82	1239.09	69.88	24.47	1226.34
	*-25%*	92.26	85.53	1288.47	$\mathbf {92.80}$	$\mathbf {87.12}$	$\mathbf {1290.15}$	74.04	1.82	1238.88	69.90	24.49	1223.55
	*+25%*	92.60	84.91	1280.35	$\mathbf {94.80}$	$\mathbf {89.05}$	$\mathbf {1285.10}$	74.04	1.82	1238.46	69.90	24.41	1217.93
	*+50%*	$\mathbf {91.66}$	$\mathbf {84.57}$	$\mathbf {1273.20}$	82.16	73.75	1252.46	74.04	1.82	1238.25	69.92	24.42	1215.15

The bold values mark the best result in each metric within each perturbation row, identifying which model performs best under that coefficient variation. At the unperturbed baseline, PPO achieves the highest accuracy (93.78%) and F1 score (87.74%), confirming the benchmark result from Table 8. Across the 16 perturbed configurations, PPO leads in both accuracy and F1 simultaneously in 10 of 16 rows (all four PLC latency perturbations, RF latency +50% , all four RF bonus perturbations except +50% , and switch penalty −50% and +50% ). In the remaining six rows, DDQN achieves higher accuracy: at all three RF latency reductions (−50% : 94.26%, −25% : 94.02%, +25% : 92.16%) and both switch penalty reductions (−25% : 92.80%, +25% : 94.80%), DDQN also wins F1 (peaking at 90.21%); in the RF bonus +50% row, DDQN wins only accuracy (90.80% vs PPO 88.14%) while PPO retains the highest F1 (80.59% vs DDQN 78.32%). Across all 16 perturbations, PPO’s own accuracy ranges from 84.96% to 93.78% and F1 from 75.22% to 87.74%, confirming broad robustness to reward coefficient changes.

The sensitivity analysis results demonstrate that the proposed PPO policy exhibits high stability and robustness against reward coefficient changes. Across most configurations, PPO maintains an accuracy above *88%* and an F1 score above *75%*, outperforming other models under almost all perturbations. Several key behavioral trends are observed. Under negative perturbations of the RF latency weight ($w_{l,\text {RF}}$) by *-50%* and *-25%*, the DDQN policy achieves higher accuracy (*94.26%* and *94.02%*, respectively) and F1 score (*90.21%* and *89.18%*) compared to PPO. This indicates that DDQN benefits from a reduced penalty on RF latency, which allows the offline Q-network updates to optimize selection ratios under high-frequency switching. However, under positive perturbations of $w_{l,\text {RF}}$, DDQN’s performance degrades (dropping to *88.76%* accuracy at *+50%*), whereas PPO maintains stable performance (*90.20%* accuracy).

In contrast, the DDPG policy shows extreme vulnerability to parameter variations. Under almost all non-baseline settings, the DDPG policy collapses, yielding an F1 score of approximately *1.8%* to *2.0%*. This collapse is attributed to the priority replay buffer implementation and deterministic continuous action clipping: variations in the reward scale shift the gradient steps outside stable bounds, causing the actor to converge to a static, single-channel policy. Similarly, Q-Learning exhibits low variability but converges to suboptimal policies, with the F1 score constrained within *22.06%* to *26.56%* across all configurations. These findings confirm that the PPO formulation is highly reliable for smart grid deployment, as the policy maintains near-optimal performance even under significant heuristic parameter changes, reducing the need for precise reward function calibration in variable field environments.

## Simulation-based network scalability and stress testing

**Fig. 15:**
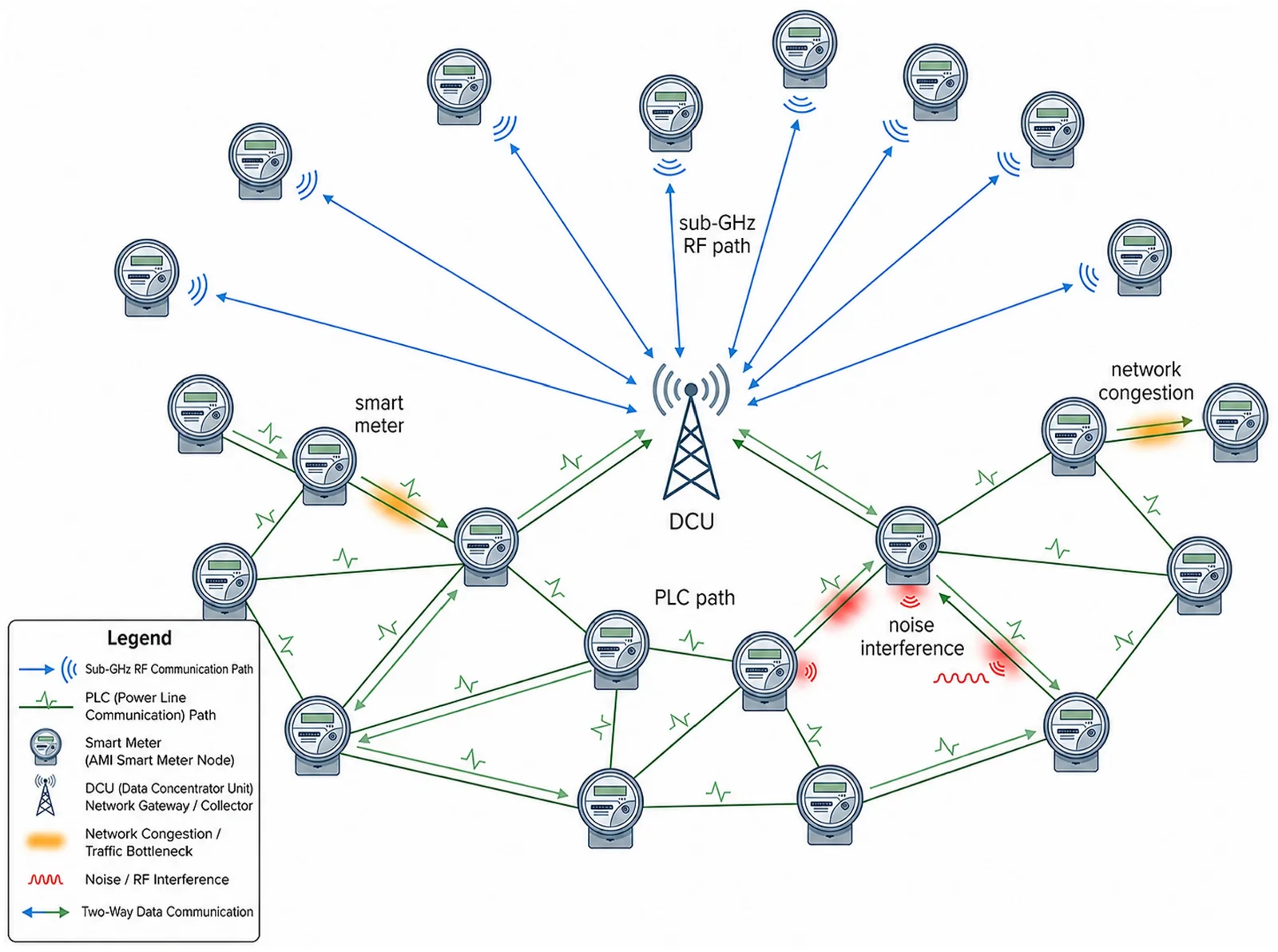
Conceptual architecture of the simulated hybrid PLC-RF smart metering network under a central DCU. *Note:* While each smart meter node is equipped with both communication interfaces, the two topologies (RF star and PLC mesh) are separated in the diagram for visual clarity.

The preceding sections validated the PPO agent’s channel selection accuracy on individual node-level decisions. This section extends the evaluation to network-scale operation through simulation, assessing whether the learned policy maintains its advantages when applied across large Advanced Metering Infrastructure (AMI) networks of 500 to 5,000 communication nodes under both normal and adversarial conditions. All results provided in this section are simulation-based validation, serving as a key stepping stone toward full operational AMI scale deployment. A conceptual diagram illustrating sub-GHz RF star and PLC mesh topologies under a single Data Concentrator Unit (DCU) is presented in Fig. 15.

### Simulation methodology

A Monte Carlo network simulation was developed to model a hierarchical AMI deployment. The simulated topology organizes *N* communication nodes into clusters of 50 nodes per Data Concentrator Unit (DCU), yielding *K = N / 50* DCU segments. This cluster size reflects the capacity of G3-PLC/PRIME concentrators in low-voltage distribution networks, which typically manage 200–300 smart meters per substation transformer^41^. The node counts of 500, 1,000, 2,000, and 5,000 were selected to represent single-concentrator through multi-concentrator Neighborhood Area Network (NAN) deployments, consistent with the scale of operational rollouts such as the Enedis Linky project^42^. Within each cluster, nodes are assigned a distance metric $d_i \in [0.1, 1.0]$ representing the normalized electrical line length or wireless separation from the local DCU. The PLC interface operates as a logical mesh with 1 to 3 routing hops based on node distance, where each additional hop adds 0.5 ms of end-to-end latency. The RF interface operates as a direct star connection to the DCU, consistent with the IEEE 802.15.4g architecture described in “RF network” section. Channel conditions for each node at each simulation time step are generated from the empirically calibrated distributions in Table 3, with distance-dependent degradation applied to SNR, latency, and packet loss.

The simulation incorporates three realistic impairment mechanisms: (1) correlated impulsive noise bursts, modeled as a 10% per-step probability of a DCU-wide event that increases PLC packet loss by 30–50% and reduces PLC SNR by 60% for all nodes in the affected cluster; (2) traffic congestion, implemented through per-DCU queue thresholds of 30 packets for PLC and 35 packets for RF, beyond which contention increases packet loss and transmission delay quadratically these thresholds correspond to approximately 70% utilization of the shared CSMA/CA medium under G3-PLC mesh overhead^43^ and the RF star topology’s channel capacity^4^; and (3) distance-dependent attenuation, where nodes farther from the DCU experience higher baseline packet loss and lower SNR. Four routing policies are compared: the trained PPO agent (using deterministic inference on the same actor network from “Reinforcement learning for channel selection” section), an Always-PLC baseline, an Always-RF baseline, and a Random (50/50) baseline. Each configuration is evaluated over 1,000 simulation time steps with a fixed random seed of 42, measuring Packet Delivery Ratio (PDR), mean and 95th-percentile end-to-end latency, throughput (delivered packets per time step), and jitter.

To clarify the nature and fidelity of this evaluation, the network-scale simulation is a custom Monte Carlo network-level model implemented directly in Python and NumPy, reusing the calibrated PLCRFNetworkEnv channel distributions described in “Simulated training environment” section, rather than an established packet-level or protocol-level network simulator such as NS-3 or OMNeT++. Within this model, PLC attenuation is represented by the empirically calibrated SNR and packet loss ranges in Table 3 combined with a distance-dependent offset, rather than a physical transmission-line or frequency-selective attenuation model, so it reproduces the aggregate statistical effect of attenuation observed in the hardware logs but not per-frequency or per-topology attenuation profiles. RF interference is likewise represented only through the calibrated baseline packet-loss and SNR distributions and the traffic congestion penalty described below, without an explicit co-channel interference or physical-layer collision model, consistent with the memoryless, interference-free channel model already described in “Simulated training environment” section. Routing overhead is approximated by a fixed latency addition of 0.5 ms per PLC mesh hop, based on the node distance to hop count mapping described above, rather than a simulated routing protocol with control-message overhead, route discovery, or topology changes. MAC contention is approximated through a per-DCU queue-threshold penalty that scales with the degree of traffic overflow beyond the 30 packet and 35 packet per-step thresholds for PLC and RF respectively, calibrated to correspond to approximately 70 percent utilization of the shared CSMA/CA medium as reported in^43^, rather than an explicit CSMA/CA backoff, collision, and retransmission simulation. This model is therefore a lightweight, empirically calibrated statistical abstraction suited to evaluating the scalability of the per-node interface-selection decision itself, which is the object of this study, rather than a substitute for protocol-level or physical-layer network simulation.

### Scalability results under normal conditions

Table 14 presents the performance of all four policies across the four network scales under normal operating conditions (no node outages, baseline traffic, impulsive noise active).

**Table 14:** Network-Scale Performance Metrics under Normal Conditions.

Node Count	Policy	PDR (%)	Latency (ms)	P95 Latency (ms)	Throughput (pkt/step)
500	Always-PLC	83.54	$\mathbf {3.16} \pm 0.56$	**4.00**	417.7
	Always-RF	85.86	*21.02 ± 2.94*	25.94	429.3
	Random	91.07	*8.84 ± 7.48*	19.84	455.3
	PPO	**92.80**	*9.52 ± 8.11*	21.77	**464.0**
1000	Always-PLC	83.32	$\mathbf {3.16} \pm 0.56$	**4.00**	833.2
	Always-RF	85.83	*21.02 ± 2.94*	25.94	858.3
	Random	91.04	*8.84 ± 7.48*	19.84	910.4
	PPO	**92.77**	*9.56 ± 8.12*	21.83	**927.7**
2000	Always-PLC	83.31	$\mathbf {3.16} \pm 0.56$	**4.00**	1666.2
	Always-RF	85.81	*21.02 ± 2.94*	25.93	1716.1
	Random	91.05	*8.85 ± 7.49*	19.85	1821.0
	PPO	**92.74**	*9.61 ± 8.15*	21.89	**1854.8**
5000	Always-PLC	83.26	$\mathbf {3.16} \pm 0.56$	**4.00**	4163.2
	Always-RF	85.84	*21.02 ± 2.94*	25.93	4292.2
	Random	91.06	*8.85 ± 7.48*	19.85	4553.0
	PPO	**92.79**	*9.56 ± 8.12*	21.81	**4639.5**

The bold values in the PPO policy achieves the highest PDR at every scale, reaching 92.80% at N = 500 and 92.79% at N = 5,000 , a variation of less than 0.1 percentage points across the entire range, confirming that PPO’s channel selection advantage is scale-invariant. PPO also achieves the highest throughput at each scale. The Always-PLC policy achieves the lowest mean and P95 latency (3.16 ms and 4.00 ms), as the PLC channel is inherently lower-latency in the calibrated distribution, but at the cost of an 83% PDR, which means roughly 1 in 6 packets is lost.

**Fig. 16:**
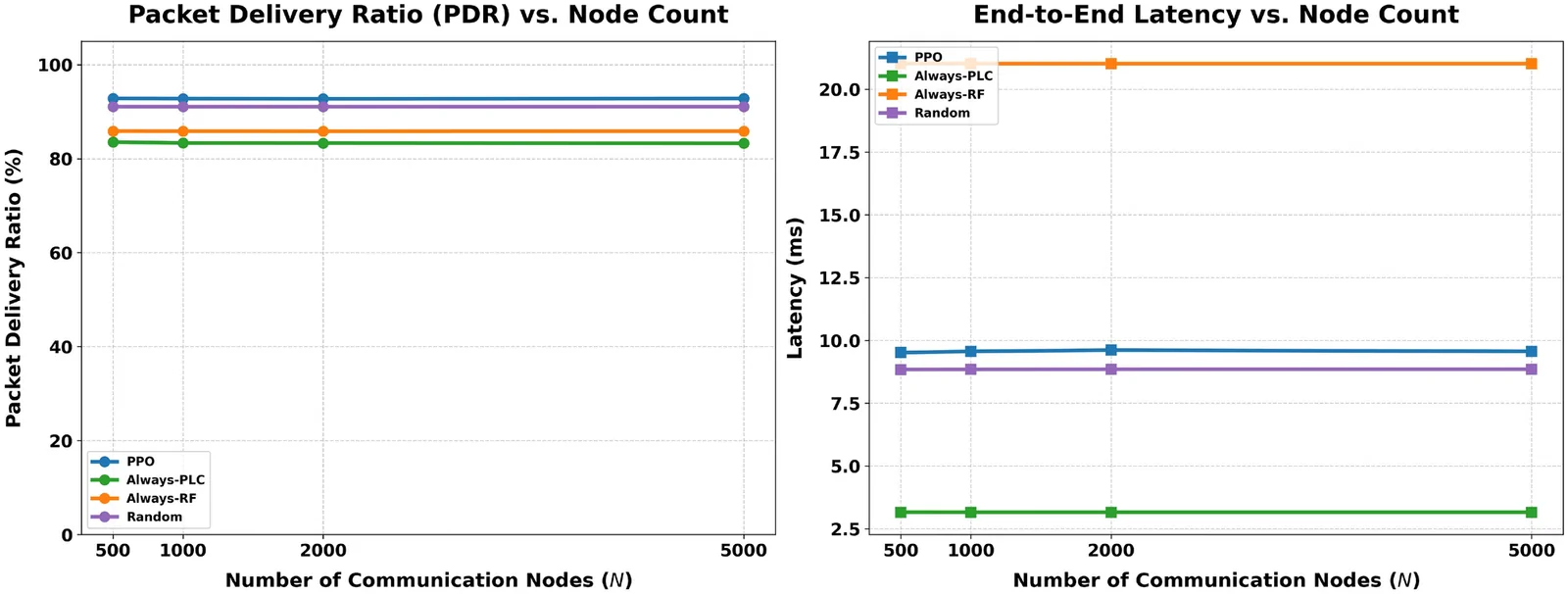
Packet delivery ratio and end-to-end latency across network scales (*N = 500* to 5, 000).

Several observations emerge from Table 14. First, the PPO policy achieves the highest PDR at every scale, delivering 92.79% of packets at $N = 5{,}000$ compared to 83.26% for Always-PLC and 85.84% for Always-RF. This represents an 11.4% relative improvement over Always-PLC and an 8.1% improvement over Always-RF. The Random baseline achieves 91.06%, indicating that even uninformed channel diversity provides substantial benefit, but PPO improves upon it by an additional 1.9 percentage points through learned, condition-aware switching. Second, the PPO policy’s performance is scale-invariant: the PDR varies by less than 0.1 percentage points across the entire 500-to-5,000 node range (92.80% to 92.79%). This stability is a direct consequence of the per-node, independent inference architecture described in “Simulation methodology” section: each node makes its channel selection decision based solely on its local channel conditions, without requiring inter-node coordination. Third, the latency profile reveals the expected trade-off: Always-PLC achieves the lowest mean latency (3.16 ms) because PLC links are inherently lower-latency in the calibrated distribution (Table 3), while Always-RF suffers from the highest latency (21.02 ms). The PPO agent’s mean latency of 9.56 ms reflects its mixed-channel strategy, which prioritizes packet delivery over latency minimization a design choice aligned with AMI reliability requirements. The throughput metric scales linearly with node count for all policies, confirming that no policy introduces systemic bottlenecks at these scales. The scalability trends are visualized in Fig. 16.

### Stress-test resilience

To evaluate robustness under adversarial conditions, a series of stress tests were conducted at the maximum scale of $N = 5{,}000$ nodes. Four categories of stress were applied: node outages (5%, 10%, 20% of nodes disabled), traffic overload (*2×*, *5×*, *10×* baseline packet rates), channel degradation (PLC SNR halved and RF packet loss doubled), and impulsive noise (comparison of clean versus noisy channels). Table 15 reports the PDR for all four policies under each scenario.

**Table 15:** Packet delivery ratio (%) under grid stress scenarios ($N = 5{,}000$).

Stress scenario	Always-PLC	Always-RF	Random	PPO
Normal (No Stress)	83.26	85.84	91.06	**92.79**
Node Outage (5%)	83.90	87.16	91.06	**92.95**
Node Outage (10%)	84.48	88.45	91.09	**93.07**
Node Outage (20%)	85.76	91.05	91.03	**93.33**
High Traffic (*2×*)	70.83	59.15	84.17	**84.45**
High Traffic (*5×*)	33.77	0.00	**54.19**	52.66
High Traffic (*10×*)	0.00	0.00	**16.83**	16.76
Channel Degradation	83.24	79.66	87.94	**89.73**
Impulsive Noise	83.27	85.85	91.04	**92.77**

The bold values mark the highest PDR in each row. Under normal and most degraded conditions the PPO policy achieves the highest PDR (e.g., 92.79% at baseline, 89.73% under channel degradation), confirming that learned adaptive switching delivers more packets than any static policy. Under extreme traffic overload (5× and 10×), Random achieves the highest PDR (54.19% and 16.83% respectively) because all deterministic policies either collapse or are constrained by single-channel saturation; in these saturated conditions no per-node selection strategy can prevent packet loss, and random diversity becomes comparably effective.

**Fig. 17:**
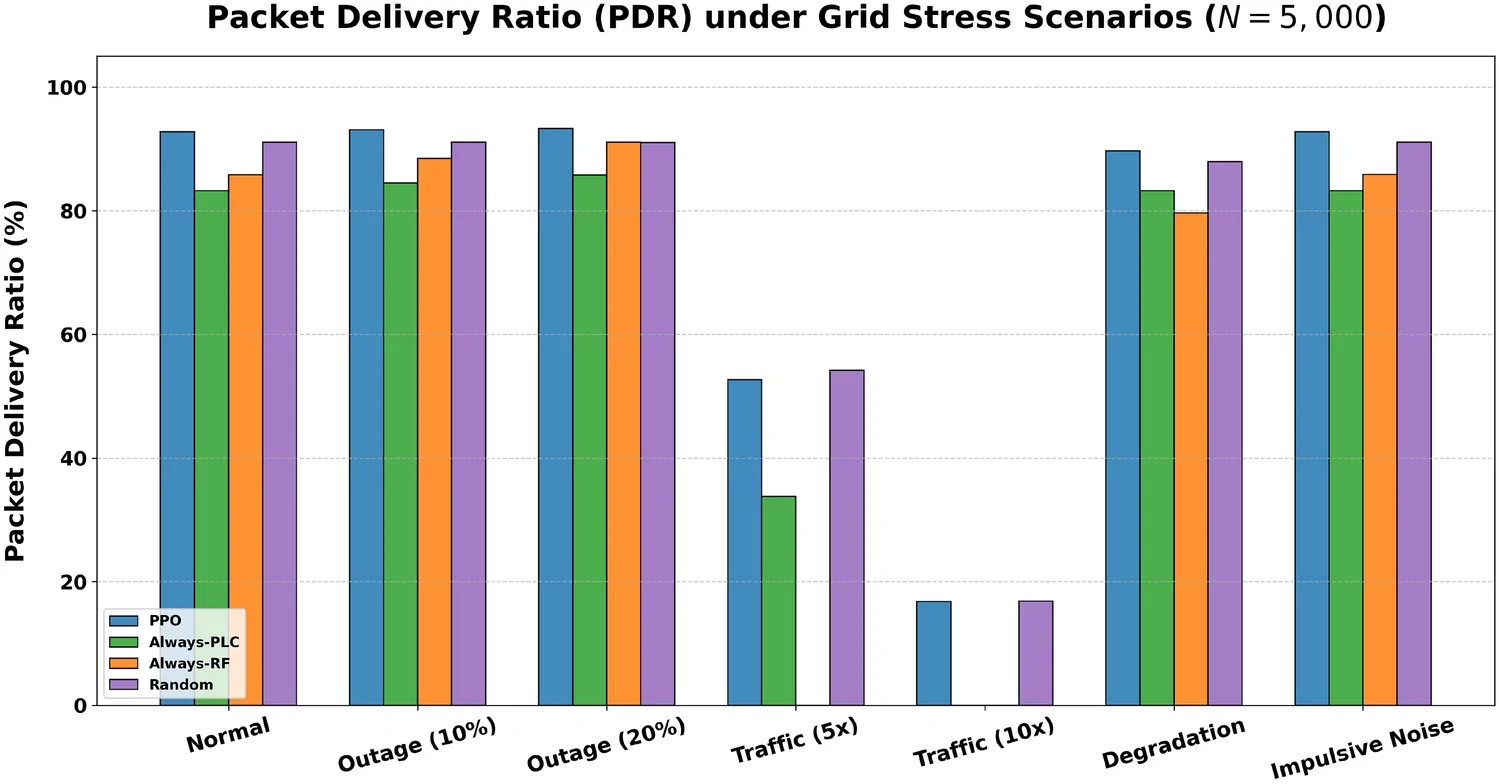
PDR comparison across stress scenarios at $N = 5{,}000$ nodes.

**Fig. 18:**
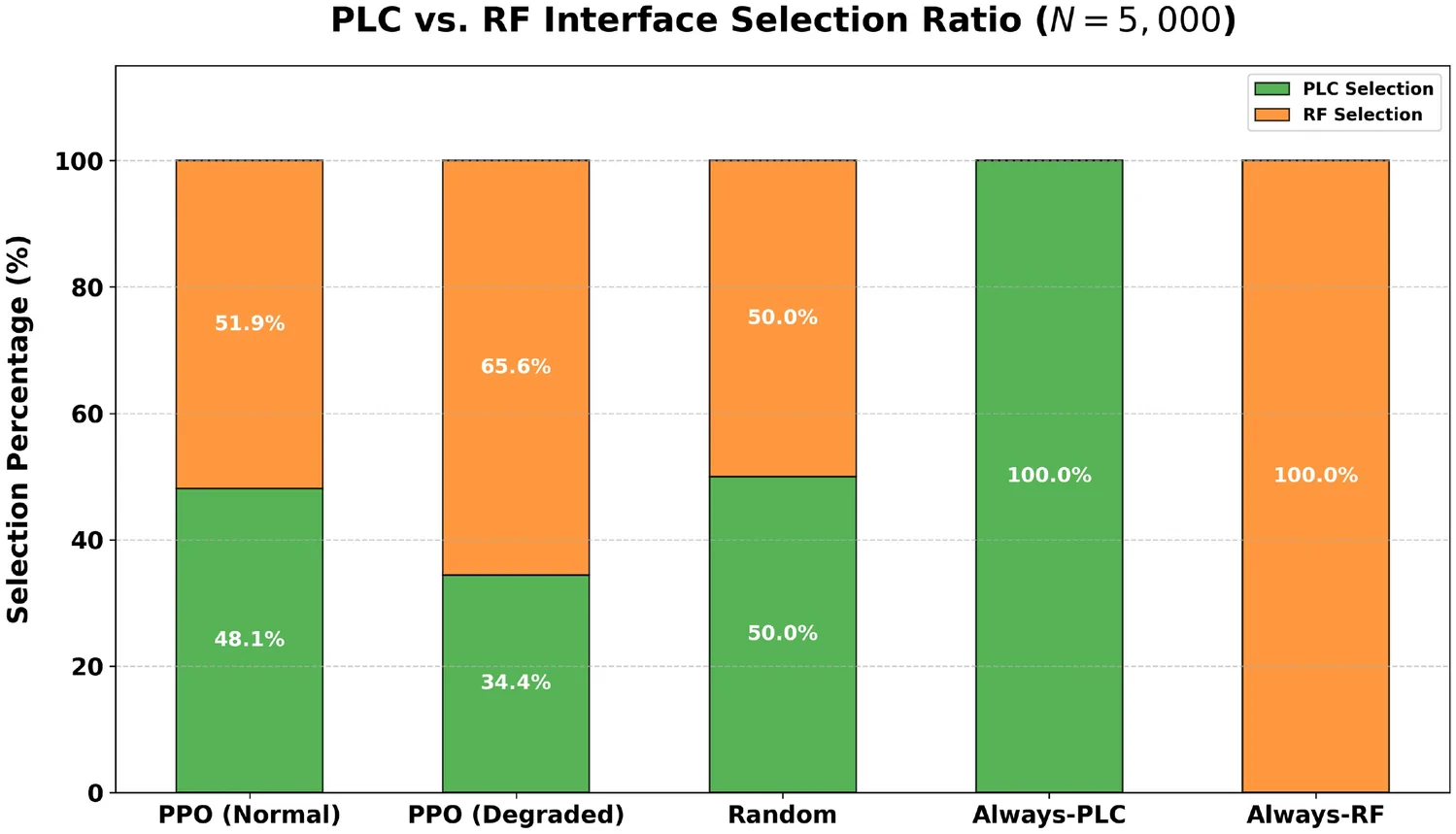
PLC vs. RF interface selection ratios under normal and degraded conditions ($N = 5{,}000$).

Under node outages of 5% to 20%, the PPO agent’s PDR actually increases slightly (from 92.79% to 93.33%) because fewer active nodes reduce per-DCU congestion. The Always-RF baseline benefits disproportionately from outages (rising from 85.84% to 91.05% at 20% outage) because RF congestion is more sensitive to node density; nonetheless, PPO maintains a consistent lead across all outage levels. Under high traffic conditions, all policies degrade substantially. At 2× load, PPO maintains 84.45% PDR while Always-RF drops to 59.15%, demonstrating the RF channel’s vulnerability to contention. At 5× load, Always-RF collapses to 0% PDR as all RF packets exceed the congestion threshold, while PPO still delivers 52.66%. At the extreme 10× load, all policies fall below 17% PDR, indicating that the simulated congestion model saturates both channels a physically realistic outcome given that both PLC and RF media share limited bandwidth. Under these extreme conditions, PPO and Random perform comparably because no per-node channel selection strategy can compensate for systemic channel saturation; addressing this would require higher-layer interventions such as rate limiting or load shedding. Under channel degradation (PLC SNR halved, RF loss doubled), PPO achieves 89.73% PDR compared to 79.66% for Always-RF, a 12.6% relative improvement. This scenario specifically stresses the RF channel, and the PPO agent responds by shifting its channel distribution: the PLC selection ratio drops from 48.1% under normal conditions to 34.4% under degradation (Fig. 18), indicating that the agent correctly increases its RF avoidance when RF link quality deteriorates. The impulsive noise scenario demonstrates that the correlated noise model has a modest but measurable impact: PPO’s PDR drops from 93.55% (clean) to 92.77% (noisy), a reduction of less than 1 percentage point, indicating resilience to bursty PLC interference. The stress-test results are visualized in Fig. 17.

### Centralized inference scalability

In a deployment architecture where PPO inference is performed centrally at the DCU or gateway level rather than on individual meter nodes, a key concern is whether the inference computation introduces a bottleneck as the number of managed nodes increases. To evaluate this, Table 16 reports the batch inference timing measured during the simulation on a GPU-equipped server (NVIDIA T4, Google Colab), where all node decisions within a time step are computed in a single batched forward pass through the PPO actor network.

**Table 16:** PPO batch inference overhead at centralized DCU level.

Node Count	Total inference time (s)	Step inference time (ms)	Amortized per-node latency (*μ*s)
500	11.45	11.45	22.90
1000	11.66	11.66	11.66
2000	**11.25**	**11.25**	5.63
5000	11.34	11.34	**2.27**

The bold value in the N = 2,000 configuration achieves the lowest total and step inference time (11.25 s), while N = 5,000 achieves the lowest amortized per-node latency (2.27 μs), since the fixed cost of a batched forward pass is shared across more nodes as batch size grows. This confirms O(1) scaling: serving 10× more nodes (N = 500 to N = 5,000 ) reduces the per-node cost by 10×, from 22.9 μs to 2.27 μs, while keeping the total inference wall-time below 11.7 s regardless of scale.

The total inference time remains constant at approximately 11.3–11.7 seconds across all scales (1,000 simulation steps), confirming that the TensorFlow batch inference operation exhibits *O*(1) scaling with respect to node count. The amortized per-node latency decreases from 22.9 *μ*s at *N = 500* to 2.27 *μ*s at $N = 5{,}000$, demonstrating favorable parallelization as the batch size grows. These results confirm that centralized PPO inference at a DCU managing up to 5,000 nodes introduces negligible computational overhead relative to the communication latencies of 1–25 ms observed on the PLC and RF links (Table 3). The feasibility of per-node edge inference on resource-constrained hardware is evaluated separately in Section Edge deployment feasibility and hardware profiling.

## Discussion

To contextualize the performance of the proposed PPO-based hybrid PLC/RF channel selection, a comparison is made with representative recent AI-based approaches in smart grid communication and operation. Table 17 summarizes the main application domain, AI method, and reported quantitative performance for each work.

**Table 17:** Quantitative comparison with recent AI-based smart grid communication approaches.

Work	Application	AI Method	Metric	Performance
This work (PPO)	Hybrid PLC/RF channel selection	PPO-based RL	Accuracy / F1 score	93.78% / 87.74%
			Avg. latency / loss	4.94 ms / 4.07%
Q-RPL^17^	AMI wireless mesh routing (network layer)	Q-learning-based routing	PDR improvement	+5–16% vs RPL variants
			Median end-to-end delay	177–286 ms
			Compliant factor	94–100%
Moussa et al.^15^	PLC node availability prediction	SVM, AdaBoost, Random Forest	Accuracy / F1 score	87% / 0.87 (AdaBoost)
			Precision / recall	0.90 / 0.86
Liu et al.^22^	Microgrid economic scheduling	DDPG	Cost reduction vs baselines	29-31% vs DQN; 17-18% vs SAC
Olojede et al.^21^	Grid fault detection	Hybrid ML/RL	Fault classification accuracy	88–93%

As shown in Table 17, the proposed PPO-based media selection model achieves 93.78% accuracy and an F1 score of 87.74% (evaluated against a reward-derived oracle on a single fixed-seed run, as described in “Reinforcement learning for channel selection” section), comparable to or slightly higher than other AI-based methods in smart grid contexts, such as the AdaBoost classifier in^15^ (87% accuracy, F1 of 0.87). Q-RPL^17^ demonstrates that reinforcement learning can significantly improve routing performance at the network layer, achieving a 5-16% increase in packet delivery ratio and high compliant factors (94-100%) in AMI wireless meshes, while the present work focuses on a complementary problem: selecting between heterogeneous PLC and RF links at each smart meter based on real-time link metrics. Within this broader landscape, where Liu et al.^22^ and Olojede et al.^21^ report strong gains in economic scheduling and fault detection accuracy using deep RL and hybrid ML/RL approaches, the proposed PPO-based framework extends the use of reinforcement learning to hybrid PLC-RF AMI communication, achieving high decision accuracy and low latency/loss without relying on large labeled datasets and operating directly on link-quality measurements.

### Scalability and deployment considerations

It is important to note that the hardware prototype comprised three RF sensor nodes and five PLC nodes, and serves as a proof-of-concept demonstration of communication feasibility and RL-based channel selection under laboratory conditions. The scalability analysis reported below is derived entirely from simulation and should not be interpreted as validated deployment performance at scale. The strong testing results of the PPO agent, achieving 93.78% accuracy (Table 8) and the lowest average packet loss (Table 9), indicate that the learned policy is suitable for real-time deployment. This is further corroborated by the simulation-based network-scale evaluation in “Simulation-based network scalability and stress testing” section, where in simulation the PPO policy maintained a PDR above 92% across topologies of 500 to 5,000 nodes with scale-invariant performance (Table 14). The deterministic on-device inference latency of 1.93 ms on an STM32 Cortex-M4 microcontroller (Table 12) is negligible relative to link-layer transmission times, confirming computational feasibility at the edge. Alternatively, as shown in Table 16, inference can be centralized at the DCU level, yielding an amortized per-node latency of 2.27–22.9 *μ*s without introducing computational bottlenecks. The main overhead is the dual-interface design, but this additional hardware cost is offset by the gain in resilience, as the hybrid PLC–RF architecture maintains connectivity in conditions where single-medium solutions are likely to fail. In terms of practical deployment cost, the dual-interface design adds the bill-of-materials cost of a second communication chipset, the LX200V50 PLC modem alongside the CC1352R RF transceiver, relative to a single-medium meter design, but removes the need for a separate cellular modem and its associated recurring data subscription fees, which is a common alternative AMI backhaul option. A detailed bill-of-materials and total cost of ownership comparison across these deployment options was outside the scope of the present study. Regarding interoperability with established AMI and grid standards, the RF interface already implements IEEE 802.15.4g at the physical and MAC layers through the TI 15.4 stack, as described in “RF network” section, so interoperability at that layer is already achieved by construction. DLMS/COSEM is the dominant international standard for smart meter data modeling and application-layer exchange, and adopting it at the Head-End System interface, in place of the current proprietary REST API and MongoDB-based backend, would allow the system to exchange meter data with third-party utility head-end systems using a standardized object model, though this has not been implemented in the current prototype. IEC 61850 is primarily used for substation automation and protection communication rather than meter-level AMI data exchange, and would become relevant mainly if the DCU to utility integration in the present architecture were extended toward broader substation automation; within the AMI-focused scope of the present work, it is not directly applicable. By reusing existing power lines and unlicensed sub-GHz spectrum instead of relying solely on cellular infrastructure, the proposed approach supports scalable AMI roll-outs with predictable long-term operational costs and aligns with the need for interoperable communication solutions in low-voltage distribution networks. Furthermore, policy robustness under reward weight variations (“Reward function sensitivity and policy robustness analysis” section) minimizes the need for high-precision coefficient tuning, which is a major deployment benefit when channel distributions drift over time.

It is important to note that the current hardware validation was conducted at laboratory scale: RF testing occurred at distances of 0.5–1.0 m, and PLC mesh nodes were spaced 1.2–2.0 m apart on the same electrical phase. A subsequent point-to-point PLC test achieved a range of approximately 9 m in a separate laboratory environment, confirming that connectivity is maintained provided the modules remain on the same electrical phase. These distances are representative of a proof-of-concept demonstration rather than an operational AMI deployment. However, the communication modules employed offer substantially greater range under field conditions. The TI CC1352R RF module, operating at sub-1 GHz frequencies with the TI 15.4 stack, supports line-of-sight ranges exceeding 1 km at 10 dBm transmit power^29^, and the LX200V50 PLC module is rated for communication distances up to 500 m over low-voltage power lines^30^. A link-budget analysis using the measured RSSI of approximately *-40* dBm at 10 dBm transmit power (Fig. 3) and assuming a receiver sensitivity of *-110* dBm yields a fade margin of approximately 70 dB, which is sufficient to accommodate path loss at distances of several hundred meters in typical urban environments. In a scaled deployment, the hierarchical architecture (sensor nodes *→* DCU *→* HES) inherently supports geographic scaling, as each DCU covers a neighborhood segment. The per-node PPO agent operates independently, requiring no inter-node coordination for channel selection, which eliminates communication overhead that would otherwise grow with network size. Routing complexity within the PLC mesh is managed by the VPN overlay and standard mesh routing protocols, and scaling to larger topologies would primarily involve adding additional DCUs rather than modifying the channel selection mechanism itself. Nonetheless, comprehensive field trials at representative AMI distances and node densities remain essential future work to validate the system under real-world propagation conditions, varying traffic loads, interference scenarios, and failure modes.

Regarding performance over longer feeders specifically, low-voltage distribution feeders extending hundreds of meters introduce frequency-selective fading and higher, more variable attenuation driven by impedance mismatches and branch topology, effects not explicitly modeled by the calibrated latency, packet loss, and SNR ranges in Table 3 or Table 4, as already noted among the limitations of the present simulation environment. Because the interface-selection mechanism observes channel quality only through these three statistical indicators rather than through the underlying physical cause of degradation, its expected response to a longer, higher-impedance feeder is a shift in the observed PLC latency, packet loss, and SNR distribution rather than a qualitatively different failure mode for the architecture itself. The closest existing evidence for this response is the channel degradation stress scenario in “Simulation-based network scalability and stress testing” section, where halving PLC SNR and doubling RF packet loss caused the PPO agent PLC selection ratio to fall from 48.1 percent to 34.4 percent, demonstrating that the learned policy shifts toward increased RF reliance as PLC channel quality deteriorates. Since RF star connectivity to a nearby DCU is largely insensitive to PLC feeder length, an analogous or stronger shift toward RF would be expected over long, high-impedance feeders in practice. Explicit validation over real distribution feeders with representative impedance profiles and impulsive noise statistics, however, has not been performed. Regarding RF specific propagation environments, the experiments reported in “RF network” section were conducted under controlled, single path indoor laboratory conditions at short range, and do not include urban multipath fading, industrial electromagnetic interference, non line of sight obstruction, or the RF co-channel interference that would arise among densely co-located smart meters sharing the same sub-GHz spectrum. As already noted in “Simulated training environment” section, the calibrated simulation environment likewise does not model multipath fading or co-channel RF interference explicitly, so the empirically observed SNR and packet loss ranges in Table 3 reflect this benign laboratory propagation environment rather than these harsher conditions. Node density effects are evaluated only at the level of the Monte Carlo network-scale simulation in “Simulation-based network scalability and stress testing” section, which models per-DCU traffic congestion and correlated impulsive noise but does not model RF physical-layer contention or interference among densely co-located transceivers. Because the interface-selection mechanism responds to any combination of degraded latency, packet loss, and SNR regardless of the underlying propagation cause, it is expected to remain functionally applicable under these conditions, but quantifying its performance under urban multipath, industrial interference, non line of sight links, and dense co-channel deployments has not been performed.

### Security considerations

Security is a critical requirement for AMI deployments operating over critical infrastructure. The proposed architecture incorporates multiple layers of security through the underlying communication stacks. The RF communication layer, implemented via the TI 15.4 stack on the CC1352R module, provides MAC-level security through AES-128 CCM* encryption and authentication as specified by the IEEE 802.15.4 standard^26^. The CC1352R hardware includes a dedicated cryptographic accelerator supporting AES 128- and 256-bit operations, which offloads encryption from the main processor and enables efficient key management with minimal computational overhead. For the PLC backhaul, the VPN overlay provides encrypted tunneling between mesh nodes. The cloud-based backend enforces TLS/SSL for all data in transit and implements role-based access control (RBAC) to restrict data visibility.

From a machine learning security perspective, the PPO agent’s observation space (latency, packet loss, SNR) could be vulnerable to adversarial perturbation through metric manipulation, where a malicious actor spoofs or jams channel measurements to bias the agent’s switching decisions. Potential attack vectors include RF jamming to artificially degrade the RF channel metrics, or injection of noise on the power line to manipulate PLC SNR readings. Mitigating such threats would require anomaly detection on the input observation space (e.g., statistical bounds checking, temporal consistency verification) and adversarial training to improve the agent’s robustness to perturbed inputs. These defensive mechanisms, along with a comprehensive threat model covering authentication, encryption overhead, and privacy implications of meter-level communication data, constitute important directions for future investigation.

Beyond adversarial perturbation of the observation space discussed above, several additional AI specific threat categories are relevant to an RL enabled communication architecture and are discussed here in relation to the specific design of the proposed system. Reward poisoning, in which an attacker corrupts the signal used to update the policy, is not a live threat against the currently deployed agent because, as established in “Edge deployment feasibility and hardware profiling” section, the deployed PPO policy is trained offline in the calibrated simulation environment and flashed as a fixed policy rather than updated online from field observations. The attack surface for reward poisoning would only become relevant if a future online or continual learning extension were introduced, at which point field derived reward signals would need to be authenticated and bounded before being used for policy updates. Model extraction is a concern specific to edge deployed machine learning, since the 64.7 KB INT8 policy reported in Table 12 is physically stored in the Flash memory of each smart meter node, some of which may be installed in physically accessible locations. Without protection, an attacker with physical access could attempt to read out the Flash contents through the microcontroller debug interface and recover the trained policy. The STM32F401RCT6 supports hardware Flash readout protection, which can be configured to disable debug port access to the Flash memory and mitigate this class of attack, and its use is recommended for field deployment though not yet enabled in the current laboratory prototype. Replay attacks, where previously captured valid frames are retransmitted to manipulate node state or channel metric readings, are addressed at the RF layer by the frame counter mechanism defined within the IEEE 802.15.4 CCM* security suite already employed by the TI 15.4 stack, which the current prototype assumes is enabled as part of the standard MAC layer security configuration described above, though explicit validation of replay resistance on the physical hardware has not been performed. False data injection targeting the meter consumption data path, as distinct from the channel metric observations discussed above, is mitigated at the application layer through the TLS protected backhaul and the role based access control already implemented in the web application, which restrict which entities may submit or modify meter readings reaching the Head End System. Finally, RL policy manipulation as a broader category subsumes several of the mechanisms above. Manipulation through crafted observations is addressed by the anomaly detection and adversarial training directions already discussed, manipulation through unauthorized model replacement is addressed by the Flash readout protection discussed above, and manipulation through a compromised update channel is currently precluded by the absence of an over the air update mechanism in the present prototype, as noted in “Edge deployment feasibility and hardware profiling” section. Introducing such a mechanism in future work would require cryptographically signed and verified model updates to prevent this attack vector from being reintroduced. Taken together, these considerations indicate that the current fixed policy, physically deployed architecture narrows the practical AI specific attack surface relative to a system employing online learning or remote model updates, while identifying concrete hardware and protocol level mitigations, namely Flash readout protection, IEEE 802.15.4 frame counters, and signed update verification, as priorities for hardening future field deployments.

### Limitations

Several limitations of the current study should be acknowledged. First, the hardware validation was conducted at laboratory scale with short communication distances and a small number of nodes (three RF sensors, five PLC nodes). While the communication modules support significantly greater ranges, the system has not been validated at operational AMI distances or node densities. Second, the physical testbed experiments remain limited to single-run validations with a small number of physical nodes. Third, the simulated environment samples channel metrics independently within calibrated bounds, which does not fully capture the temporal correlations, impulsive noise distributions, and cyclic load-driven packet loss patterns characteristic of real PLC channels. Fourth, the current state representation is limited to three features per channel (latency, packet loss, SNR) and does not capture interference variability or temporal traffic patterns beyond the congestion model evaluated in “Simulation-based network scalability and stress testing” section. Fifth, the network-scale simulation evaluates interface selection in isolation and does not model interaction with higher-layer routing protocols, which may introduce additional coordination overhead in multi-hop scenarios. Sixth, the sensitivity analysis utilized a static, one-factor-at-a-time (OAT) parameter variation methodology. In real-world environments, joint multi-parameter changes and time-varying reward coefficient interactions may occur, which are not modeled by single-parameter sensitivity sweeps. Seventh, the communication performance evaluation is confined to the three metrics directly observable by the RL agent (latency, packet loss, SNR) plus PDR at the network scale. Extended metrics including throughput, goodput, BER, jitter, outage probability, channel utilization, retransmission overhead, and per-node energy consumption require packet-level hardware instrumentation beyond what the current testbed exposes. Eighth, the framework as evaluated assumes that PLC and RF channel observations at a given node are affected by independent random processes, and the network-scale correlated impulsive noise mechanism in “Simulation-based network scalability and stress testing” section models correlation only within the PLC medium at DCU scale. It does not explicitly model common-mode failures that could simultaneously degrade both media, such as a local transformer or DCU power outage that removes electrical continuity for PLC transmission and also interrupts power to a co-located RF gateway. In the present hierarchical architecture, both the PLC mesh and the RF star topology terminate at the same central DCU, so a DCU-level outage would interrupt connectivity for all nodes in that segment regardless of which channel the per-node policy selects; this is an architectural single point of failure that per-node interface selection cannot mitigate on its own. The Node Outage stress test in “Simulation-based network scalability and stress testing” section evaluates the case of individual nodes going fully offline and shows that aggregate network delivery is not degraded by such outages, but it does not specifically model a shared upstream event correlated across multiple nodes and both channels simultaneously. Furthermore, the simulated channel model represents degraded conditions as bounded latency, packet loss, and SNR values rather than an explicit binary unavailable state for each channel, so it cannot currently distinguish a fully unavailable medium from a severely degraded but nominally present one. Modeling DCU-level common-mode outages explicitly, together with a discrete channel-unavailable state and backup power or cellular fallback considerations, would strengthen future evaluations of this failure mode.

### Future research

Future work will prioritize field trials at representative AMI distances and node densities to validate the system under real-world propagation conditions. The simulation environment will be enhanced with time-correlated channel models and coupled with network-layer routing protocols to evaluate end-to-end performance. Future comparative studies should also include representative threshold-based heuristic switching rules commonly used in hybrid PLC-RF systems. The current prototype deploys only the trained PPO actor for fixed, offline inference; deploying the full agent, including the critic and a rolling buffer of field transitions, could instead enable on-device continual learning that adapts to long-term link drift, at the cost of a larger memory footprint than the current INT8 deployment. Validation against independent hardware traces or an established network simulator such as NS-3 or OMNeT++ would also strengthen the evaluation, since the current hardware logs were retained only as calibration ranges (Table 3) rather than raw traces suitable for direct policy replay. Additional research directions include federated learning^24^ for privacy-preserving distributed updates, lightweight on-device training via TinyML frameworks^25^, hardware consolidation onto a single-chip solution, and online reward weight tuning through meta-reinforcement learning to reduce manual calibration across diverse grid topologies.

## Conclusion

This paper presented a hybrid PLC–RF communication system designed to enhance the reliability and adaptability of smart metering networks within Advanced Metering Infrastructure. The architecture was validated using physical LX200V50 PLC modules configured as a mesh network and TI CC1352R RF modules operating in a star topology under the TI 15.4 stack, demonstrating link rerouting, fault tolerance, and reliable end-to-end data transmission to a central Data Concentrator Unit. A cloud-based web application was integrated for real-time monitoring of power consumption, network connectivity, and active communication paths. To enable intelligent channel selection, a Proximal Policy Optimization (PPO) reinforcement learning agent was implemented and benchmarked against tabular Q-Learning, DDQN, and DDPG, dynamically switching between PLC and RF based on real-time latency, packet loss, and SNR observations. Evaluated over 5, 000 deterministic test cases against a reward-derived oracle, the PPO agent achieved an accuracy of *93.78%* and an F1 score of *87.74%*, attaining the highest single-run performance among all evaluated agents, with statistically significant advantages over Q-Learning and, for F1 score, over DDPG. Multi-seed training across ten distinct initializations confirmed superior convergence stability with an accuracy standard deviation of only *8.74%*, mitigating the policy collapse risks observed in DDQN. Edge AI profiling on an Arm Cortex-M4 microcontroller platform demonstrated that full INT8 quantization compresses the model by *2.36×* to a footprint of 64.7 KB with a deterministic inference latency of 1.93 ms, preserving *>99.9%* of the baseline accuracy. A reward function sensitivity analysis under 16 coefficient perturbations confirmed the robustness of the learned policy across varying weight configurations. Finally, a simulation-based network-scale evaluation across 500 to 5, 000 nodes confirmed scale-invariant performance with a Packet Delivery Ratio above *92%* and per-node batch latencies of 2.27–22.9 *μ*s, while stress tests under node outages, traffic overload, and channel degradation demonstrated consistent resilience advantages over static baselines. These results collectively provide simulation-based evidence of the viability of AI-driven adaptive communication for resilient, large-scale smart grid infrastructures.

## Data Availability

The data generated supporting the findings of this paper are available within the article. Specifically, the empirical data rangesused for the calibration of the simulated environment are provided in Table 3. Further information regarding the customsimulation environment or the raw hardware logs is available from the corresponding author upon reasonable request.
